# Prevalence of antibiotic prescribing in COVID-19 patients in China and other low- and middle-income countries during the pandemic (December 2019—March 2021): a systematic review and meta-analysis

**DOI:** 10.1093/jac/dkad302

**Published:** 2023-10-26

**Authors:** Wenjuan Cong, Hung-Yuan Cheng, Beth Stuart, Binjuan Liu, Yunyi Tang, Yi Wang, Nour AIhusein, Hexing Wang, Amit Manchundiya, Helen Lambert

**Affiliations:** Department of Population Health Sciences, Bristol Medical School, University of Bristol, 39 Whatley Road, Bristol BS8 2PS, UK; Department of Population Health Sciences, Bristol Medical School, University of Bristol, 39 Whatley Road, Bristol BS8 2PS, UK; Centre for Evaluation and Methods, Wolfson Institute of Population Health, Faculty of Medicine and Dentistry, Queen Mary University of London, Yvonne Carter Building, 58 Turner Street, London E1 2AB, UK; Department of Population Health Sciences, Bristol Medical School, University of Bristol, 39 Whatley Road, Bristol BS8 2PS, UK; Department of Population Health Sciences, Bristol Medical School, University of Bristol, 39 Whatley Road, Bristol BS8 2PS, UK; Key Laboratory of Public Health Safety of Ministry of Education, School of Public Health, Fudan University, 130 Dongan Road, Shanghai 200243, China; Department of Population Health Sciences, Bristol Medical School, University of Bristol, 39 Whatley Road, Bristol BS8 2PS, UK; Key Laboratory of Public Health Safety of Ministry of Education, School of Public Health, Fudan University, 130 Dongan Road, Shanghai 200243, China; Department of Population Health Sciences, Bristol Medical School, University of Bristol, 39 Whatley Road, Bristol BS8 2PS, UK; Department of Population Health Sciences, Bristol Medical School, University of Bristol, 39 Whatley Road, Bristol BS8 2PS, UK

## Abstract

**Objectives:**

Low- and middle-income countries (LMICs) are particularly vulnerable to the threat of antimicrobial resistance (AMR). Use of antibiotics to treat COVID-19 patients during the pandemic may have contributed to increasing the AMR burden, but systematic evidence is lacking.

**Methods:**

We searched Web of Science, EMBASE, PubMed, China National Knowledge Infrastructure (CNKI) and VIP databases from 1 December 2019 to 31 March 2021. Interventional and observation studies across all settings that reported antibiotic use in at least 10 COVID-19 patients were included. We restricted publications to English and Chinese languages. Screening and data extraction were undertaken by at least two independent reviewers. Results were synthesized using random-effects meta-analyses. Subgroup analyses and meta-regression were used to explore heterogeneities. This review was registered with PROSPERO (CRD42021288291).

**Results:**

We included 284 studies involving 210 611 participants in 19 countries. The antibiotic prescribing rates (APRs) in COVID-19 inpatients were 71.7% (95% CI 66.7%–76.5%) in China and 86.5% (77.1%–93.9%) in other LMICs, respectively. APR was lower in mild/moderate cases in China [66.9% (57.9%–75.4%) compared with 91.8% (71.4%–100%) in other LMICs]. High APRs were found among pregnant women and the elderly in China. Disparities in APRs of other patient groups were identified. In studies reporting bacterial infections, the prevalence was 17.3% (10.0%–25.9%) in China and 24.9% (0.1%–68.8%) in other LMICs. Several antibiotics on the WHO ‘Watch’ and ‘Reserve’ lists were prescribed frequently in LMICs.

**Conclusions:**

Inappropriate antibiotic use and high prevalence of antibiotic prescribing were found in COVID-19 inpatients in many LMICs.

## Introduction

Antimicrobial resistance (AMR) is a threat to global health and development; it occurs when bacteria change over time and no longer respond to antibiotics, making infections harder to treat and increasing the risk of severe illness and death.^1^ Low- and middle-income countries (LMICs) are disproportionately affected by AMR, due to their prevailing high levels of poverty, high burden of infectious diseases, poor regulation and limited surveillance of antimicrobial use, a lack of access to quality antimicrobials, and constrained health resources.^2,3^ An estimated 4.95 million deaths were associated with AMR globally in 2019, with 1.27 million deaths attributed to AMR.^4^ Of these, sub-Saharan Africa and South Asia had the highest mortalities (23.5 deaths per 100 000 and 21.5 deaths per 100 000, respectively) attributable to AMR, compared with other regions.^4–6^

The COVID-19 pandemic may have augmented the AMR crisis through widespread and inappropriate antibiotic prescribing in COVID-19 patients during the early and middle stages of the pandemic.^7–10^ Our previous scoping reviews found that during the first phase of the pandemic (December 2019–June 2020), 82.3% of COVID-19 patients in published studies were prescribed at least one antibiotic, and antibiotics were prescribed for COVID-19 patients regardless of severity of illness.^7^ In the subsequent 9 months (June 2020–March 2021), the antibiotic prescribing rate for COVID-19 patients declined to 39.7% and there was a substantial reduction in antibiotic prescribing (from 75.4% to 15.5%) for mild and moderate patients.^9^ The scale of antibiotic use in LMICs is generally under-represented in studies due to difficulties in capturing this complex information and use of antibiotics in COVID-19 patients in these settings may well be under-reported. In May 2020, WHO recommended against antibiotic use for patients with mild COVID-19.^11^ However, the national treatment guidelines for COVID-19 in Ghana, Kenya, Uganda, South Africa, Zimbabwe, Botswana, Liberia, Ethiopia and Rwanda were still recommending antibiotic use in mild and even suspected COVID-19 patients in June 2021. Some countries frequently used broad-spectrum antibiotics, which are on the WHO ‘Watch’ and ‘Reserve’ lists, to treat patients with COVID-19.^12^ Additionally, during the pandemic most LMICs reported limited ability to work with AMR partnerships, reduced availability of nursing, medical and public health staff for antibiotic stewardship and also decreased funding for AMR activities.^13^ These factors together aggravate the existing burden of AMR in LMICs and increase vulnerability to the threat of AMR.

However, there are limited data on antibiotic prescribing and bacterial infections in COVID-19 patients from most LMICs, with the exception of China.^7,9^ Additionally, antibiotic prescribing patterns and bacterial infections in COVID-19 patients from specific groups such as elderly patients, pregnant women, paediatric patients and patients with existing health conditions may differ from those of the general population. Therefore, in this study we aim to describe the prevalence of antibiotic prescribing and bacterial infection in COVID-19 patients in LMICs over the first 15 months of the pandemic, before effective vaccines and antiviral drugs started to become available.

## Materials and methods

This systematic review is reported in line with the Preferred Reporting Items for Systematic Reviews and Meta-Analyses (PRISMA)^14^ and the PRISMA checklist can be found in Table S1 (available as Supplementary data at *JAC* Online). The protocol is registered under the international prospective register of systematic reviews (PROSPERO; CRD42021288291).

### Searches and selection criteria

We searched the following electronic databases: Web of Science, EMBASE, PubMed, China National Knowledge Infrastructure (CNKI) and VIP databases from December 2019 to March 2021. The search strategy can be found in Table S2.

We included observational studies, such as cohort (prospective or retrospective), cross-sectional, case–control, case series and controlled trials that did not evaluate antibiotic use as an intervention. Inclusion was restricted to studies with sample sizes of at least 10 patients. We also did not set a limit on age although we accidentally put a limit to adult patients in the protocol registered with PROSPERO. There was no restriction in populations and healthcare settings if studies were conducted in LMICs. The definition of LMICs is based on the Development Assistance Committee (DAC) list of official development assistance (ODA) recipients from the Organisation for Economic Co-operation and Development (OECD).^15^ We included research articles that reported COVID patients (confirmed by RT–PCR or clinician diagnosis) receiving antibiotic treatment during the course of their illness and excluded reviews, editorials, case reports, trial protocols, clinical guidelines, case series with fewer than 10 patients, and conference abstracts. We restricted publications to English and Chinese languages.

The primary outcome was the percentage of patients prescribed antibiotics. Secondary outcomes included details of prescribed antibiotics, discharge rates, mortality and percentage of patients with confirmed bacterial infections.

### Screening and data extraction

Titles and abstracts were screened initially by W.C., N.A., B.L. and Y.T., and full texts of potentially eligible references were retrieved to determine the eligibility for data extraction against the inclusion and exclusion criteria. Study selection was conducted in duplicate independently and disagreement was resolved by consensus.

We designed and piloted bespoke data extraction sheets and extracted the following data from each study report: study characteristics (including country, region, settings, study period, sample sizes, study design) and population characteristics (including age, gender, comorbidities, COVID-19 severity, length of hospital stay). Data extraction was conducted by W.C., N.A., B.L., Y.W. and Y.T. and double-checked by W.C. and A.M. Disagreements were resolved by consensus. The lead author (W.C.) participated throughout the screening and data extraction stages to increase inter-rater reliability. Studies were cleaned and reviewed to minimize double counting by noting similar study locations and periods. A risk-of-bias assessment was not conducted due to lacking valid tools.

### Data synthesis

We summarized antibiotic prescribing rates by China and other LMICs since the majority of studies were from China, as noted in previous reviews,^7,9^ due to its primary position in the COVID-19 outbreak, disease control and response measures during the pandemic.

We stratified studies into two groups: general population and distinct populations. For studies reporting on patients from the general population, we further stratified results by setting (inpatients, outpatients and mixed settings) since settings are associated with disease severity and antibiotic prescribing. Studies reporting on specific populations, such as patients with pre-existing conditions or with specific clinical considerations (paediatric patients, pregnant women, elderly patients) were grouped separately since pre-existing conditions may influence antibiotic prescribing. As the severity of COVID-19 illness (mild/moderate and severe/critical cases) serves as a proxy for complications and clinical decision-making regarding antibiotic use, we also presented the results separately to elucidate antibiotic prescribing patterns. A similar approach applied to other health outcomes, discharge rates and mortality among patients prescribed with antibiotics.

Prevalence of antibiotic prescribing in each study was calculated as a percentage of patients receiving antibiotics. The 95% CIs for each percentage were estimated using the exact method.^16^ If there were two or more studies, a meta-analysis was performed using Freeman–Tukey double-arcsine transformation and inverse variance meta-analysis approaches in random-effects models via the *meta* package in R (version 4.2.2).^17^ Although we planned to measure the heterogeneity (between-study variation) using the *I*^2^ statistic^18^ in the protocol, *I*^2^ has often been found to be extremely high (> 90%) and unsuitable for meta-analyses of prevalence due to the large sample sizes and number of studies.^19^ Instead, we reported meta-analysis results complemented by descriptive statistics (mean/median and IQR). In addition, we applied additional subgroup analyses and meta-regression to elucidate the underlying factors affecting antibiotic prescribing when there were at least 10 studies. We used categorical covariates, types of study design and (for studies conducted in China only) provinces in subgroup analyses and continuous covariates, mean percentage of males and mean length of hospital stay in meta-regression analyses. We could not use other covariates such as age, continent, OECD category and study period due to poor reporting or highly skewed data. We also utilized a meta-regression approach to determine the association between antibiotic prescribing and secondary outcomes. Graphs were made using the ggplot2 package in R.^20^ Other results, including study characteristics and details of antibiotics prescribed, were tabulated and summarized narratively.

## Results

**Figure 1.**
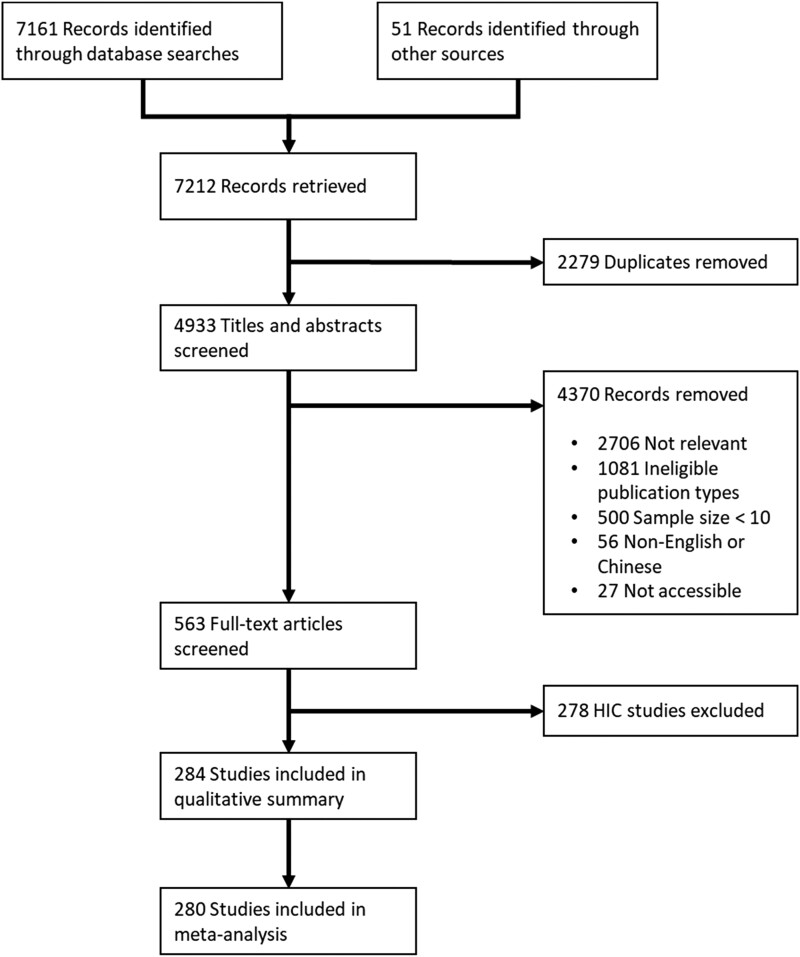
Study selection.

We screened 4933 titles and abstracts from 7212 retrieved references after removing irrelevant studies and duplicates (Figure 1). There were 563 references in the full-text screening stage. After assessing eligibility, the data from 284 studies were extracted for qualitative summary, of which 280 studies were included in the meta-analysis (see Table S3 for characteristics of studies). Overall, 210 611 COVID-19 patients were included in the meta-analysis for antibiotic prescribing (Table 1). Most studies (*k* = 221 studies) were from China and 59 were from other LMICs, including Bahrain (*k* = 1), Bangladesh (*k* = 3), Brazil (*k* = 12), Egypt (*k* = 1), India (*k* = 5), Iran (*k* = 14), Iraq (*k* = 1), Malaysia (*k* = 1), Mexico (*k* = 4), North Macedonia (*k* = 1), Pakistan (*k* = 2), Peru (*k* = 1), Philippines (*k* = 1), Romania (*k* = 2), Russia (*k* = 1), Serbia (*k* = 1), Turkey (*k* = 7) and Vietnam (*k* = 1). Most studies were cohorts (*k* = 234), followed by controlled trials (*k* = 19). Two hundred and seventy studies focused on hospitalized COVID-19 patients, while a study in a mixed setting had the most patients (*n* = 136 855).^21^ The majority of studies recruited their patients from the general population (*k* = 214). Only a few studies focused on specific populations, such as paediatric patients (*k* = 13), pregnant women (*k* = 6), elderly people (*k* = 3) and patients with pre-existing conditions (*k* = 45). Two hundred and fifty studies indicated that COVID-19 patients were recruited during the early stages of the pandemic, from December 2019 to June 2020. Out of these, 209 studies exclusively reported COVID-19 patients enrolled between December 2019 and March 2020, while 11 studies exclusively focused on patients recruited between April 2020 and June 2020.

**Table 1.** Summary of studies included in the meta-analysis for antibiotic use

	China	Other LMICs
Total number of studies (patients)	221 (60 185)	59 (150 426)
Sample size, median (range)	109 (10–5419)	91 (10–136 855)
Proportion male (%), median range	52 (0–83)^a^	58 (39–80)^b^
Patient type		
General	170 (46 568)^c^	43 (148 858)
Paediatric patients	6 (478)	7 (481)
Pregnant women	6 (277)	—
Elderly patients	3 (444)	—
AIDS	1 (17)	—
Asthma and COPD	1 (961)	—
Cancer	2 (240)	1 (181)
COPD	1 (1048)	—
Diabetes	8 (3731)	2 (763)
Digestive symptoms	1 (204)	—
Epilepsy	1 (30)	—
GI disease	3 (1489)	1 (24)
Hypertension	3 (897)	—
Kidney transplant	—	3 (62)
Kidney disease	7 (2453)	—
Liver injury	4 (745)	—
NAFLD	1 (280)	—
Psychiatric patients	1 (25)	—
Stroke	1 (27)	—
Thrombocytopenia	1 (271)	—
Skin lesions	—	1 (10)
Zinc deficiency	—	1 (47)
Setting		
Inpatients	218 (59 014)	52 (12 734)
Outpatients	1 (61)	2 (65)
Mixed	2 (1110)	5 (137 627)
Design		
Case–control	2 (306)	—
Case report/series	5 (74)	4 (54)
Cohort	204 (58 211)	39 (146 629)
Cross-sectional	2 (430)	5 (1317)
Controlled trials	8 (1164)	11 (2426)

GI, gastrointestinal; NAFLD, non-alcoholic fatty liver disease.

^a^ *k* = 216 studies.

^b^ *k* = 53 studies.

^c^Total number of studies (patients).

**Figure 2.**
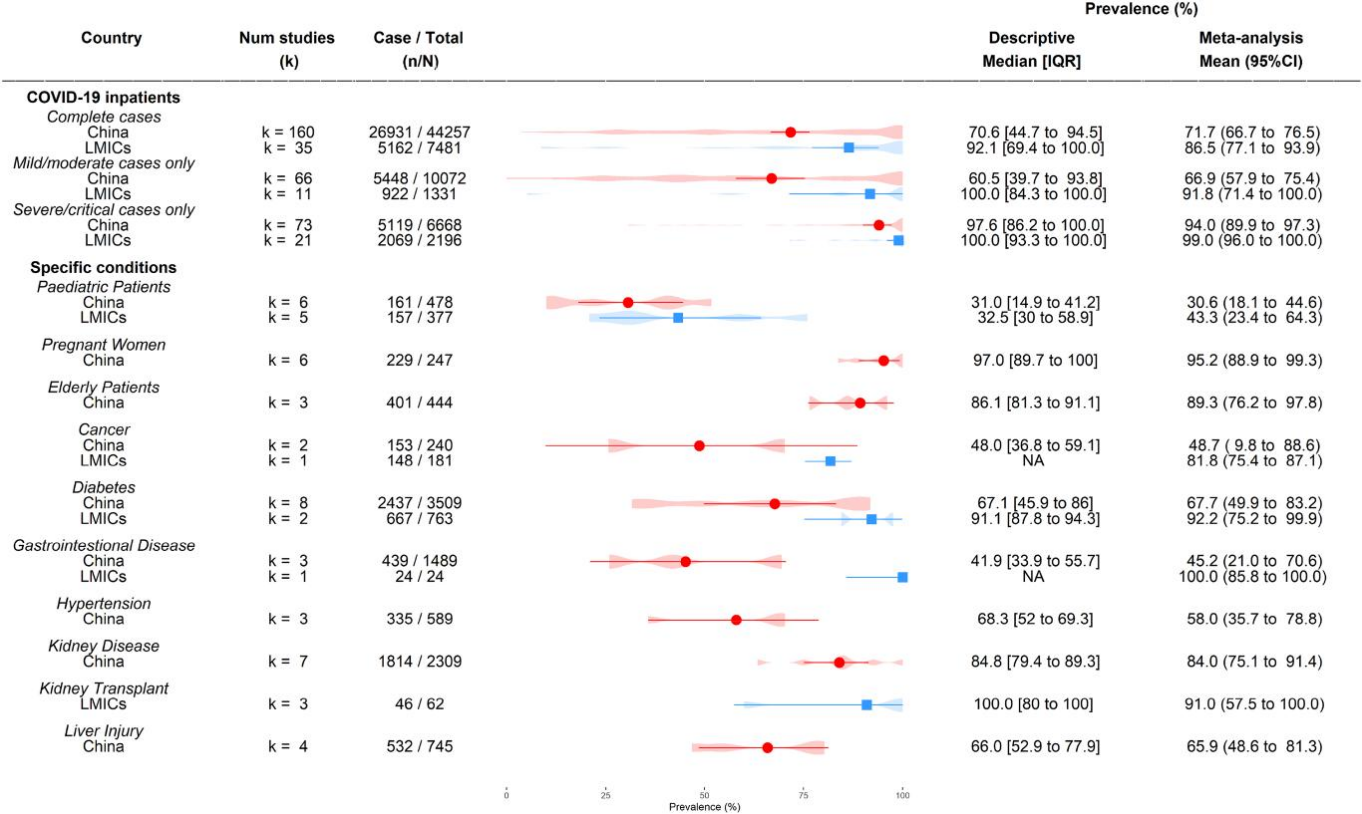
Prevalence of antibiotic prescribing in COVID-19 inpatients in China and other LMICs. Meta-analysis results of health outcomes presented by points (red circle: China; blue square: other LMICs) and error bars (95% CIs). The violin plots underneath show the detailed distribution of the prevalence of corresponding health outcomes from the included studies. This figure appears in colour in the online version of *JAC* and in black and white in the print version of *JAC*.

One hundred and sixty studies from China and 35 studies from other LMICs reported the prevalence of antibiotic prescribing for COVID-19 inpatients (Figure 2) in general populations. The overall antibiotic prescribing rates were 71.7% (95% CI 66.7%–76.5%) in China and 86.5% (77.1%–93.9%) in other LMICs, respectively. The prescribing rate was lower in mild/moderate cases in China [66.9% (57.9%–75.4%); *k* = 66)] than in the other LMICs [91.8% (71.4%–100.0%); *k* = 11]. However, both country groups had similar prescribing rates in severe/critical cases [94.0% (89.9%–97.3%); *k* = 73 in China, and 99.0% (96.0%–100.0%); *k* = 21 in other LMICs] (Figure S1).

The proportion of male patients and average length of hospital stay were not associated with antibiotic use in hospitalized patients in LMICs (Table S4). In China, although certain provinces such as Wuhan [where the pandemic began: 81.8% (75.6%–87.2%); *k* = 75] and Guangdong [73.4% (52.8%–89.7%)] had much higher prescribing rates than other provinces, significant heterogeneities were still observed in the subsets in subgroup analysis (Table S5). The antibiotic prescribing rates did not differ by the type of study design, though apart from cohorts the numbers of other study types were limited (Table S5).

There were only a few studies reported in outpatient and mixed settings. For outpatients, the antibiotic prescribing rates were 32.8% (21.3%–46%; *k* = 1) in China and 100% (93.5%–100%; *k* = 1) in other LMICs. In mixed settings, the rates were 53.2% (40.1%–66.0%; *k* = 1) in China and 48.8% (6.9%–85.5%; *k* = 3) in other LMICs.

In the populations with specific considerations (Figure 2), prescribing rates for paediatric patients were slightly lower in China [30.6% (18.1%–45.6%); *k* = 6] compared with other LMICs [43.3% (23.4%–64.3%); *k* = 5]. The only studies found reporting antibiotic prescribing rates in pregnant woman [95.2% (88.9%–99.3%); *k* = 6] and elderly patients [89.3% (76.2%–97.8%); *k* = 3] were from China. In terms of patients with pre-existing conditions, antibiotic prescribing rates differed considerably between China and other LMICs in patients with cancer, diabetes and gastrointestinal disease (Figure 2). For example, in patients with cancer, the rates were 48.7% (9.8%–88.6%; *k* = 2) in China and 81.8% (75.4%–87.1%; *k* = 1) in other LMICs. Similarly, in patients with diabetes, the rates were 67.7% (49.9%–83.2%; *k* = 8) in China and 92.2% (75.2%–99.9%; *k* = 2) in other LMICs (Figure S2 for other patient groups).

Mortality and discharge rates in COVID-19 patients prescribed antibiotics are presented in Tables S6 and S7. In general, mortality rates in China were much lower than in other LMICs, while discharge rates presented the opposite trend. Using random-effects meta-regression, mortality and discharge rates were associated with the prevalence of antibiotic use in China (*P* < 0.001 and *P* < 0.001, respectively). However, they only accounted for 14.9% and 9.2% of the prevalence, respectively. On the other hand, neither mortality nor discharge rates were associated with the prevalence of antibiotic use in other LMICs (*P* = 0.619 and *P* = 0.147, respectively).

**Figure 3.**
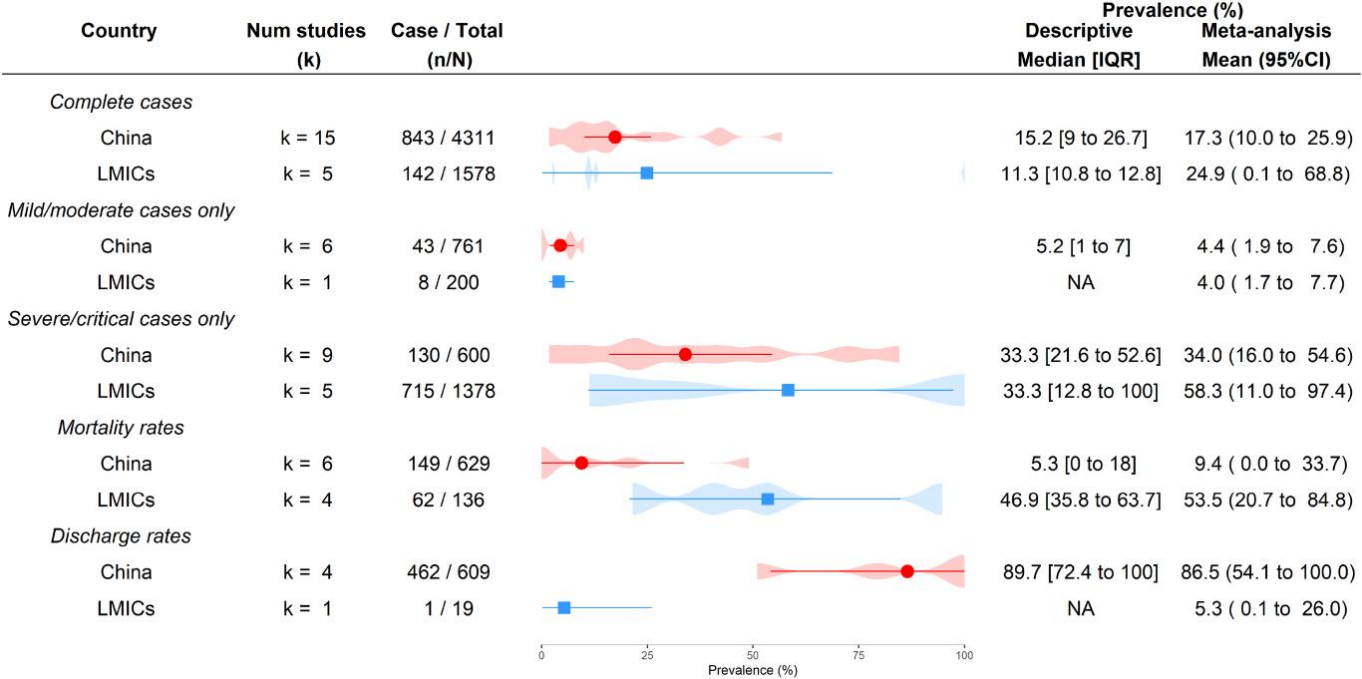
Prevalence of bacterial infections in COVID-19 patients in China and other LMICs. Meta-analysis results of health outcomes presented by points (red circle: China; blue square: other LMICs) and error bars (95% CIs). The violin plots underneath show the detailed distribution of the prevalence of corresponding health outcomes from the included studies. This figure appears in colour in the online version of *JAC* and in black and white in the print version of *JAC*.

There were 25 studies reporting bacterial infection rates, of which 20 studies recruited patients from the general population (Figure 3). The prevalence of bacterial infections was 17.3% (95% CI 10.0%–25.9%) in China and 24.9% (0.1%–68.8%) in other LMICs. Although bacterial infection rates were similar in both China and other LMICs, the rate in other LMICs [58.3% (11.0%–97.4%); *k* = 5] was higher than the rate in China [34.0% (16.0%–54.6%); *k* = 9] in severe/critical cases. Six studies reported COVID-19 patients with coinfections (4 in China and 2 from other LMICs) and 12 studies reported COVID-19 patients with secondary infection (8 in China and 4 from other LMICs) (Table S8). Only 10 studies reported resistant pathogens in COVID-19 patients and the frequently reported resistant pathogens in COVID-19 patients were *Escherichia coli*, *Klebsiella pneumoniae* and *Acinetobacter baumannii*.

**Figure 4.**
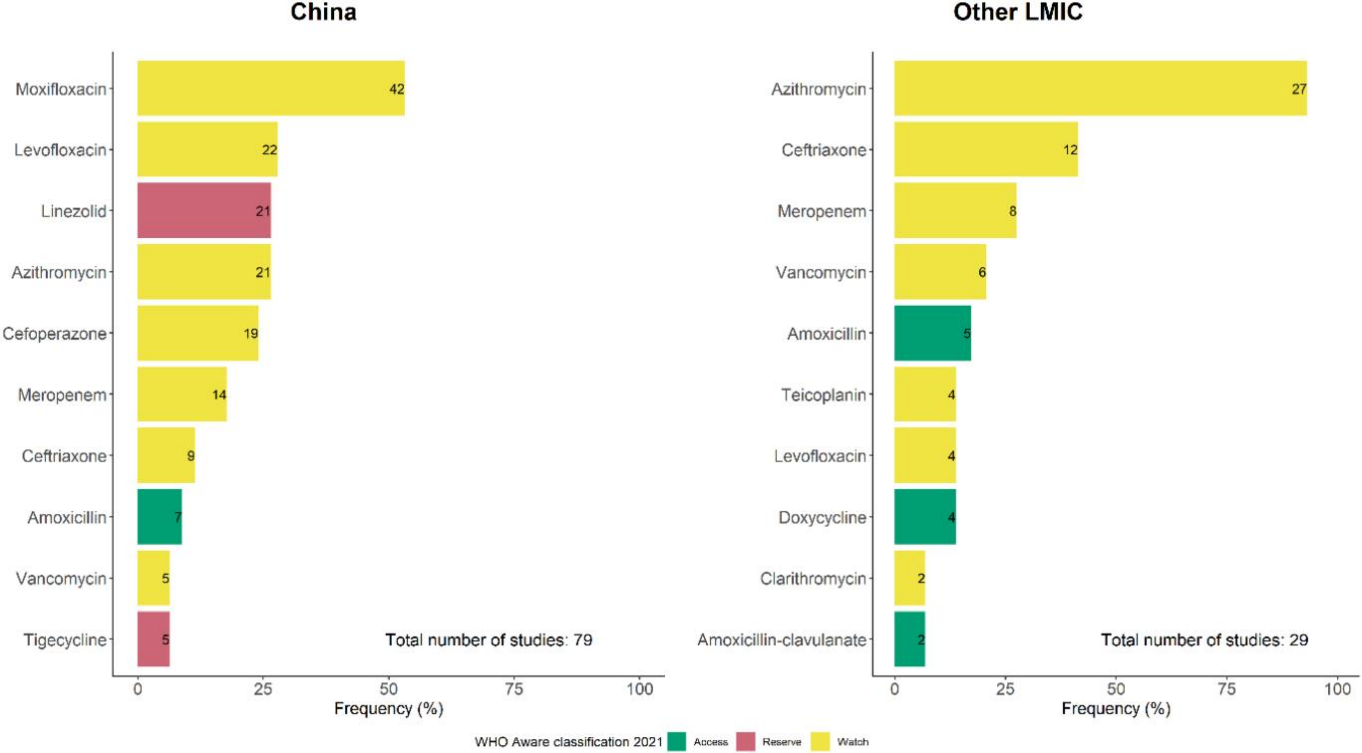
Frequently prescribed antibiotics in COVID-19 patients in China and other LMICs. This figure appears in colour in the online version of *JAC* and in black and white in the print version of *JAC*.

Seventy-nine studies from China and 29 studies from other LMICs reported types of antibiotics prescribed in COVID-19 patients. The most frequently prescribed antibiotic in China was moxifloxacin, followed by levofloxacin and linezolid, whereas azithromycin, ceftriaxone and meropenem were the most commonly used antibiotics in COVID-19 inpatients in other LMICs. According to the 2021 WHO AWaRe classification of antibiotics, the top 10 frequently prescribed antibiotics in China included two ‘Reserve’ antibiotics, seven ‘Watch’ antibiotics and one ‘Access’ antibiotic.^22^ In other LMICs, there were eight ‘Watch’ antibiotics and two ‘Access’ antibiotics in the top 10 most commonly prescribed antibiotics (Figure 4).

## Discussion

Our review and analysis of antibiotic prescribing rates for COVID-19 patients in China and other LMICs from December 2019 to March 2021 show that the scale of antibiotic use in COVID-19 patients in these settings remains concerning. First, high prevalence of antibiotic prescribing was observed in COVID-19 patients regardless of severity and in certain distinct populations, including pregnant women and elderly patients. Secondly, prevalence of bacterial infection in COVID-19 patients was much higher in our included studies from low- and middle-income settings than the ∼10% previously reported from global reviews.^23–26^ Thirdly, studies in China reported lower mortality rates, lower bacterial infection rates and higher discharge rates in COVID-19 inpatients, compared with studies in other LMICs. Fourthly, in terms of types of antibiotics prescribed, studies in China reported more frequent use of ‘last-resort’ (Reserve) antibiotics than studies in other LMICs. Finally, it is noteworthy that MDR pathogens, including *E. coli*, *K. pneumoniae* and *A. baumannii*, which were already prevalent in LMICs before the COVID-19 pandemic,^27,28^ continued to exhibit a concerning prevalence in COVID-19 patients within LMICs.

The high prevalence of antibiotic prescribing in COVID-19 patients in LMICs may in part be associated with relatively high bacterial infection rates compared with studies from high-income country (HIC) settings, especially in severe/critical cases. Due to limited surveillance and infection control practices in most LMICs, patients in ICUs have higher risk of acquiring bacterial infections in LMICs compared with HICs.^29^ However, overall rates of bacterial infection in COVID-19 patients in this review were still fairly low and cannot fully justify the high prevalence of antibiotic prescribing. In addition, the widespread use of ‘Watch’ and even (in China) ‘Reserve’ antibiotics found in this review implies an urgent need for wide dissemination of antibiotic prescribing guidelines for COVID-19 patients to clinicians and renewed promotion of antibiotic stewardship activities in LMICs to support prudent use of antibiotics and limit the development of antibiotic resistance.

The lower mortality rates, bacterial infection rates and high discharge rates seen in COVID-19 inpatients in China could result from the specific disease control measures taken in that setting. Stringent ‘Zero COVID’ restrictions were implemented in China from the start of the pandemic, and regardless of severity, all diagnosed patients had to be hospitalized and managed in specific hospitals. The larger numbers of hospitalized mild/moderate patients resulting from this policy probably accounts for the reduced mortality and elevated discharge rates in studies from China compared with other LMICs, such as India^30^ and Brazil,^31^ where patients were not hospitalized unless their symptoms had worsened significantly.

Our results are consistent with findings from a recently released WHO report on antibiotic prescribing in hospitalized COVID-19 patients globally. Antibiotic use in hospitalized COVID-19 patients was stable and surprisingly high (>90%) in Africa at the beginning of the pandemic until the summer of 2021, whereas during the same period in west European regions, antibiotic prescribing rates in mild and moderate COVID-19 inpatients were very high in the beginning of the pandemic, but subsequently largely declined.^32^

This is the first paper to systematically explore prevalence of antibiotic prescribing and bacterial infection in COVID-19 patients in LMICs by illness severity, geographic region (Wuhan, Hubei and other regions in China) and patient type. The number of COVID-19 patients included in this review was very large, especially for China. However, there are some limitations. First, although we tried to search all possible studies from other LMICs utilizing several electronic databases, the geographic coverage is still limited to 19 countries. Certain countries, especially in Africa, were not included in this review. This may result from the language restriction or from under-representation of LMICs in published health research in general. Second, extreme results and variations were commonly seen in the reported outcomes, resulting in high heterogeneities. Although we have exhausted meta-regression and subgroup analyses to investigate the key modifiers, the heterogeneities were likely to reflect the multifaceted nature of prescribing in clinical practice since most studies included in this review were not designed to investigate antibiotic prescribing in COVID-19 patients. Finally, this review only captures antibiotic prescribing in LMICs over the first 15 months of the pandemic (December 2019 to March 2021). We acknowledge that these findings could not be generalized to the current situation. As different LMICs have had substantially differing timelines in the COVID-19 crisis, studies from countries that experienced the pandemic later are under-represented in this review. However, it is important to note that the introduction of effective vaccines and antiviral drugs has significantly altered the landscape of COVID-19 treatment since the time period covered in this review, allowing healthcare facilities to better allocate resources and provide more effective patient care. This may help to reduce the unnecessary use of antibiotics in treating COVID-19.

In conclusion, antibiotic prescribing rates in LMICs were generally high in hospitalized COVID-19 patients, regardless of severity of illness, in the first 15 months of the pandemic. Higher antibiotic prescribing was observed in specific COVID-19 patient groups, especially elderly patients and pregnant women. The prevalence of bacterial infection in COVID-19 patients in LMICs was higher than in studies of COVID-19 patients from HICs. While the guidance for treating COVID-19 and prescribing antibiotics has been updated to ensure the appropriate management of bacterial infections, it remains crucial for LMICs to establish comprehensive training curricula. These curricula are essential to support clinicians to understand best practices for antibiotic use, risks and health implications associated with unnecessary antibiotic prescribing.

## Supplementary Material

dkad302_Supplementary_Data

## Data Availability

All data extraction forms, statistical analytic codes and any other materials used in the review are available on reasonable request.

## Supplementary data

Figures S1 and S2 and Tables S1 to S8 are available as Supplementary data at *JAC* Online.
