# Supplementary material for: Prevalence of antibiotic prescribing in COVID-19 patients in China and other low- and middle-income countries during the pandemic (December 2019—March 2021): a systematic review and meta-analysis
Source: J Antimicrob Chemother. 2023 Oct 26;78(12):2787–94. doi: 10.1093/jac/dkad302 (PMC10689912; doi:10.1093/jac/dkad302)
Supplement: dkad302_Supplementary_Data [file dkad302_supplementary_data.docx]

Supplementary materials

Table of Contents

[Table S1. PRISMA 2020 checklist 2](#_Toc143812422)

[Table S2. Search strategy 4](#_Toc143812423)

[Table S3. Characteristics of studies 5](#_Toc143812424)

[Figure S1. Additional forest plots 27](#_Toc143812425)

[Table S4. Meta-regression results 29](#_Toc143812426)

[Table S5. Subgroup analysis 29](#_Toc143812427)

[Figure S2. Forest plots of antibiotic use in COVID-19 patients with pre-existing conditions 31](#_Toc143812428)

[Table S6. Mortality rates of COVID-19 patients and patients with specific conditions 34](#_Toc143812429)

[Table S7. Discharge rates of COVID-19 inpatients and patients with specific conditions 35](#_Toc143812430)

[Table S8. Summary of studies included for bacterial infection analysis 36](#_Toc143812431)

## Table S1. PRISMA 2020 checklist

| **Section and Topic** | **Item #** | **Checklist item** | **Location where item is reported** |
| --- | --- | --- | --- |
| **TITLE** | | |  |
| Title | 1 | Identify the report as a systematic review. | Page 1 |
| **ABSTRACT** | | |  |
| Abstract | 2 | See the PRISMA 2020 for Abstracts checklist. | Page 2 |
| **INTRODUCTION** | | |  |
| Rationale | 3 | Describe the rationale for the review in the context of existing knowledge. | Page 3 |
| Objectives | 4 | Provide an explicit statement of the objective(s) or question(s) the review addresses. | Page 3 |
| **METHODS** | | |  |
| Eligibility criteria | 5 | Specify the inclusion and exclusion criteria for the review and how studies were grouped for the syntheses. | Page 4 |
| Information sources | 6 | Specify all databases, registers, websites, organisations, reference lists and other sources searched or consulted to identify studies. Specify the date when each source was last searched or consulted. | Page 4 |
| Search strategy | 7 | Present the full search strategies for all databases, registers and websites, including any filters and limits used. | Table S2 |
| Selection process | 8 | Specify the methods used to decide whether a study met the inclusion criteria of the review, including how many reviewers screened each record and each report retrieved, whether they worked independently, and if applicable, details of automation tools used in the process. | Page 4 |
| Data collection process | 9 | Specify the methods used to collect data from reports, including how many reviewers collected data from each report, whether they worked independently, any processes for obtaining or confirming data from study investigators, and if applicable, details of automation tools used in the process. | Page 4 |
| Data items | 10a | List and define all outcomes for which data were sought. Specify whether all results that were compatible with each outcome domain in each study were sought (e.g. for all measures, time points, analyses), and if not, the methods used to decide which results to collect. | Page 4 |
|  | 10b | List and define all other variables for which data were sought (e.g. participant and intervention characteristics, funding sources). Describe any assumptions made about any missing or unclear information. | Page 4 |
| Study risk of bias assessment | 11 | Specify the methods used to assess risk of bias in the included studies, including details of the tool(s) used, how many reviewers assessed each study and whether they worked independently, and if applicable, details of automation tools used in the process. | NA |
| Effect measures | 12 | Specify for each outcome the effect measure(s) (e.g. risk ratio, mean difference) used in the synthesis or presentation of results. | NA |
| Synthesis methods | 13a | Describe the processes used to decide which studies were eligible for each synthesis (e.g. tabulating the study intervention characteristics and comparing against the planned groups for each synthesis (item #5)). | Page 4-5 |
|  | 13b | Describe any methods required to prepare the data for presentation or synthesis, such as handling of missing summary statistics, or data conversions. | Page 4-5 |
|  | 13c | Describe any methods used to tabulate or visually display results of individual studies and syntheses. | Page 4-5 |
|  | 13d | Describe any methods used to synthesize results and provide a rationale for the choice(s). If meta-analysis was performed, describe the model(s), method(s) to identify the presence and extent of statistical heterogeneity, and software package(s) used. | Page 5 |
|  | 13e | Describe any methods used to explore possible causes of heterogeneity among study results (e.g. subgroup analysis, meta-regression). | Page 5 |
|  | 13f | Describe any sensitivity analyses conducted to assess robustness of the synthesized results. | NA |
| Reporting bias assessment | 14 | Describe any methods used to assess risk of bias due to missing results in a synthesis (arising from reporting biases). | NA |
| Certainty assessment | 15 | Describe any methods used to assess certainty (or confidence) in the body of evidence for an outcome. | NA |
| **RESULTS** | | |  |
| Study selection | 16a | Describe the results of the search and selection process, from the number of records identified in the search to the number of studies included in the review, ideally using a flow diagram. | Page 5 & Figure 1 |
|  | 16b | Cite studies that might appear to meet the inclusion criteria, but which were excluded, and explain why they were excluded. | Page 5 & Figure 1 |
| Study characteristics | 17 | Cite each included study and present its characteristics. | Table S3 |
| Risk of bias in studies | 18 | Present assessments of risk of bias for each included study. | NA |
| Results of individual studies | 19 | For all outcomes, present, for each study: (a) summary statistics for each group (where appropriate) and (b) an effect estimate and its precision (e.g. confidence/credible interval), ideally using structured tables or plots. | Page 5-6 |
| Results of syntheses | 20a | For each synthesis, briefly summarise the characteristics and risk of bias among contributing studies. | Page 5-7 |
|  | 20b | Present results of all statistical syntheses conducted. If meta-analysis was done, present for each the summary estimate and its precision (e.g. confidence/credible interval) and measures of statistical heterogeneity. If comparing groups, describe the direction of the effect. | Page 5-7 |
|  | 20c | Present results of all investigations of possible causes of heterogeneity among study results. | Page 5-7 |
|  | 20d | Present results of all sensitivity analyses conducted to assess the robustness of the synthesized results. | NA |
| Reporting biases | 21 | Present assessments of risk of bias due to missing results (arising from reporting biases) for each synthesis assessed. | NA |
| Certainty of evidence | 22 | Present assessments of certainty (or confidence) in the body of evidence for each outcome assessed. | NA |
| **DISCUSSION** | | |  |
| Discussion | 23a | Provide a general interpretation of the results in the context of other evidence. | Page 7-8 |
|  | 23b | Discuss any limitations of the evidence included in the review. | Page 8 |
|  | 23c | Discuss any limitations of the review processes used. | Page 8 |
|  | 23d | Discuss implications of the results for practice, policy, and future research. | Page 8 |
| **OTHER INFORMATION** | | |  |
| Registration and protocol | 24a | Provide registration information for the review, including register name and registration number, or state that the review was not registered. | Page 2, Page 4 |
|  | 24b | Indicate where the review protocol can be accessed, or state that a protocol was not prepared. | PROSPERO; CRD42021288291 |
|  | 24c | Describe and explain any amendments to information provided at registration or in the protocol. | Page 4 |
| Support | 25 | Describe sources of financial or non-financial support for the review, and the role of the funders or sponsors in the review. | Page 9 |
| Competing interests | 26 | Declare any competing interests of review authors. | Page 9 |
| Availability of data, code and other materials | 27 | Report which of the following are publicly available and where they can be found: template data collection forms; data extracted from included studies; data used for all analyses; analytic code; any other materials used in the review. | Page 9 |

## Table S2. Search strategy

The following databases: Web of Science, EMBASE, PubMed and two Chinese academic databases (CNKI & VIP) were searched to identify relevant studies from Dec 2019 up to Mar 2021. No limits were set on the country where study was conducted, and we excluded any studies not available in English or Chinese due to our review team capacity.

The search terms were: ((“COVID-19” or “SARS-CoV-2” or “Coronavirus disease 2019” or “severe acute respiratory syndrome coronavirus-2”) and ((“antibiotic prescribing” or “antibiotic use” or “antibiotic*”) or “antimicrobial *” or “antimicrobial therapy” or “antimicrobial resistance” or “antimicrobial stewardship”)).

**Web of Science:**

All Fields = (COVID-19 and antibiotic*) or (SARS-CoV-2 and antibiotic*) or (Coronavirus disease 2019 and antibiotic*) or (severe acute respiratory syndrome coronavirus-2 and antibiotic*)

All Fields = (COVID-19 and antimicrobial*) or (SARS-CoV-2 and antimicrobial*) or (Coronavirus disease 2019 and antimicrobial*) or (severe acute respiratory syndrome coronavirus-2 and antimicrobial*)

**PubMed:**

(COVID-19 or SARS-CoV-2 or Coronavirus disease 2019 or severe acute respiratory syndrome coronavirus-2) and antimicrobial*

(COVID-19 or SARS-CoV-2 or Coronavirus disease 2019 or severe acute respiratory syndrome coronavirus-2) and antibiotic*

**Embase**:

(antibiotic* or antibiotic prescribing or antimicrobial resistance or antibacterial*) and (severe acute respiratory syndrome coronavirus-2 or COVID-19 or SARS-CoV-2 or Coronavirus disease 2019).

**CNKI & VIP**:

新型冠状病毒或新冠肺炎或2019冠状病毒或COVID-19; 抗生素使用或抗菌药物使用或抗菌药物管理或抗生素耐药性

## Table S3. Characteristics of studies

| **Study** | **Design** | **Study period** | **Country** | **Population size** | **Settings^*^** | **% Male** | **Age^#^** |
| --- | --- | --- | --- | --- | --- | --- | --- |
| Prata-Barbosa et al^1^ | Cohort | 1/3-31/5 2020 | Brazil | 79 | IN | 54.0% | 4.0 |
| Wu et al^2^ | Cohort | 20/1-27/2 2020 | China (multiregions) | 65 | IN | 59.5% | 6.0 |
| Yang et al^3^ | Cohort | 17/1-5/2 2020 | China (Hunan) | 40 | IN | 57.5% | 45.0 |
| Chen et al^4^ | Cohort | 15/1-15/3 2020 | China (Zhejiang) | 32 | IN | 65.6% | 9.5 |
| Yu et al^5^ | Cohort | 1/1-12/2 2020 | China (Hubei) | 23 | IN | 0.0% | 32.3 ± 6.2 |
| Jothimani et al^6^ | Cohort | 17/5-27/5 2020 | India | 47 | IN | 61.5% | 34.0 |
| Gu et al^7^ | Cohort | 25/1-20/2 2020 | China (Jiangsu) | 66 | IN | 53.0% | 43.0 |
| Cao et al^8^ | Cohort | 3/1-1/2 2020 | China (Hubei) | 102 | IN | 52.0% | 54.0 (37.0-67.0) |
| Li et al^9^ | Cohort | 5/2-27/2 2020 | China (Anhui) | 49 | IN | 57.1% | 45.0 (32.0-60.0) |
| Feng et al^10^ | Cohort | 1/2-1/3 2020 | China (Hubei) | 134 | IN | 48.5% | 45.0 |
| Zhang et al^11^ | Cohort | 25/1-29/2 2020 | China (Sichuan) | 78 | IN | 56.4% | 46.5 |
| Fan et al^12^ | Cohort | 20/1-15/3 2020 | China (Liaoning) | 55 | IN | 54.5% | 46.8 |
| Lv et al^13^ | Cohort | 1/1-27/1 2020 | China (Hubei) | 101 | IN | NA | NA |
| Chowdhary et al^14^ | Case series | 1/4-31/7 2020 | India | 15 | IN | NA | NA |
| Tang et al^15^ | RCT + Randomised parallel group | 11/2-29/2 2020 | China (Multiregions) | 150 | IN | 30.0% | 48.0 |
| Gao et al^16^ | Cohort | 20/1-17/3 2020 | China (Beijing) | 62 | MIX | 55.0% | 48.3 |
| Qiu et al^17^ | Cohort | 6/2-11/2 2020 | China (Zhejiang) | 64 | IN | 59.4% | 48.5 |
| Bastug et al^18^ | Cohort | 13/3-30/3 2020 | Turkey | 191 | IN | 56.0% | 49.0 |
| Wang et al^19^ | Cohort | 20/1-25/3 2020 | China (Multiregions) | 156 | IN | 52.6% | 51.0 |
| Soltani et al^20^ | Cohort | 1/3-15/4 2020 | Iran | 19 | IN | NA | NA |
| Ding et al^21^ | Cohort | 20/1-15/3 2020 | China (Shanghai) | 311 | IN | 52.0% | 51.0 |
| Liu et al^22^ | Cohort | 24/1-17/2 2020 | China (Beijing) | 80 | IN | 46.0% | 51.2 |
| Deng et al^23^ | Cohort | 24/1-10/3 2020 | China (Chongqing) | 61 | OUT | 41.0% | 54.8 |
| Hu et al^24^ | Cohort | 27/1-28/3 2020 | China (Hubei) | 1254 | IN | 48.9% | 56.0 |
| Zhou et al^25^ | Cohort | As of 25/2 2020 | China (Shandong) | 508 | IN | 58.9% | NA |
| Bustos-Cordova et al^26^ | Cohort | 1/4-31/7 2020 | Mexico | 50 | IN | 70.0% | 56.6 |
| Yuan et al^27^ | Cohort | 22/1-31/5 2020 | China (Hubei) | 14 | IN | 52.1% | NA |
| Abad et al^28^ | Cohort | 1/3-31/3 2020 | Philippines | 40 | IN | 57.5% | 60.5 |
| Li et al^29^ | Cohort | 1/1-20/2 2020 | China (Hubei) | 134 | IN | 56.0% | 61.0 |
| Camelo-Filho et al^30^ | Cross-sectional | 15/3-31/5 2020 | Brazil | 15 | IN | 40.0% | 61.3 |
| Chen et al^31^ | Cohort | 30/1-26/2 2020 | China (Hubei) | 52 | IN | 59.6% | 62.0 |
| Zhang et al^32^ | Cohort | 28/1-9/3 2020 | China (Hubei) | 440 | IN | 47.0% | 63.0 |
| Wang et al^33^ | RCT + Randomised parallel group | 6/2-12/3 2020 | China (Hubei) | 236 | IN | 59.3% | 65.0 |
| Cheng et al^34^ | Cohort | 28/1-11/2 2020 | China (Hubei) | 701 | IN | 52.4% | 66.0 |
| Escobar et al^35^ | Cohort | 11/3-31/3 2020 | Peru | 14 | IN | 78.6% | 73.4 |
| Dang et al^36^ | Cohort | 20/1-20/2 2020 | China (Hubei) | 17 | IN | 70.6% | 88.0 |
| Sun et al^37^ | Cohort | 20/1-15/2 2020 | China (Beijing) | 55 | IN | 56.4% | 44.0 (34.0-56.0) |
| Fu et al^38^ | Cohort | 26/1-2/3 2020 | China (Yunnan) | 33 | IN | 45.0% | NA |
| Zhang et al^39^ | Cohort | 1/1-18/3 2020 | China (Multiregions) | 365 | IN | 48.2% | 46.8 ± 15.5 |
| Du et al^40^ | Cohort | 26 Jan to 5 Mar 2020 | China (Hubei) | 114 | IN | 63.0% | 7.1 |
| Feng et al^41^ | Case series | 31 Jan to 26 Feb 2020 | China (Henan) | 11 | IN | 72.8% | NA |
| Zhang et al^42^ | Cohort | As of 16/3 2020 | China (Hubei) | 31 | IN | 58.1% | 62.3 ± 14.4 |
| Teich et al^43^ | Cohort | 26/2-25/3 2020 | Brazil | 72 | MIX | 56.9% | 39.9 ± 13.6 |
| Abrishami et al^44^ | Case series | 1/2-30/4 2020 | Iran | 12 | IN | 75.0% | 47.7 ± 1.4 |
| Rabha et al^45^ | Cohort | 1/3-30/6 2020 | Brazil | 115 | MIX | 57.4% | 2.0 (0.9-8.0) |
| Vee et al^46^ | Cohort | 9/3-15/4 2020 | Malaysia | 247 | IN | 69.6% | 28.0 (20.0-45.0) |
| Merza et al^47^ | Cohort | 18/3-7/4 2020 | Iraq | 15 | IN | 60.0% | 28.1 ± 16.4 |
| Zeng et al^48^ | Cohort | As of 16/2 2020 | China (Hubei) | 16 | IN | 0.0% | 31.0 ± 3.8 |
| Liu et al^49^ | Cohort | 20/1-10/2 2020 | China (Hubei) | 15 | IN | 0.0% | 32.0 ± 5.0 |
| Xu et al^50^ | Cohort | 15/1-15/3 2020 | China (Hubei) | 34 | IN | 0.0% | 32.2 ± 4.7 |
| Yu et al^51^ | Cohort | 17/2-6/3 2020 | China (Hubei) | 214 | IN | 57.5% | 47.0 |
| Wei et al^52^ | Cohort | 18/1-2/3 2020 | China (Hubei) | 43 | IN | NA | NA |
| Wei et al^53^ | Cohort | 18/1-31/1 2020 | China (Hubei) | 12 | IN | 25.0% | 35.0 (33.0-41.0) |
| Kosugi et al^54^ | Cross-sectional | 25/3-30/4 2020 | Brazil | 253 | IN | 47.0% | 36.0 (31.0-44.0) |
| Wang et al^55^ | Cohort | 25/12/2019-15/2/2020 | China (Hubei) | 118 | IN | 41.7% | 36.6 ± 7.4 |
| Ye et al^56^ | Cohort | 22/1-29/1 2020 | China (Zhejiang) | 47 | IN | 47.0% | NA |
| Huang et al^57^ | Cohort | 20/1-10/6 2020 | China (Shanghai) | 135 | IN | 54.3% | 37.0 (25.0–53.0) |
| Liu et al^58^ | Cohort | 17/1-29/2 2020 | China (Hunan) | 87 | IN | 48.3% | 37.0 (17.0-59.0) |
| Ye et al^59^ | Cohort | 8/1-10/2 2020 | China (Hubei) | 55 | IN | 34.5% | 37.0 (22.0-67.0) |
| Nguyen et al^60^ | Cohort | 6/3-15/4 2020 | Vietnam | 44 | IN | 52.0% | 37.5 ± 16.8 |
| Xia et al^61^ | Cohort | 31/12/2019-8/2/2020 | China (Hubei) | 54 | IN | 50.0% | 56.5 |
| Wang et al^62^ | Cohort | 20/1-9/2 2020 | China (Anhui) | 125 | IN | 56.8% | 38.8 ± 13.8 |
| Zhong et al^63^ | Cohort | 1/1-14/2 2020 | China (Hubei) | 49 | IN | 12.2% | NA |
| IIic et al^64^ | Cohort | 20/3-22/4 2020 | Serbia | 107 | MIX | NA | 39.1 ± 11.4 |
| Lin et al^65^ | Cohort | 9/2-26/2 2020 | China (Hainan) | 457 | IN | 39.6% | 47.8 ± 11.0 |
| Huang et al^66^ | Cohort | 21/12/2019-11/3/2020 | China (Hubei) | 51 | IN | 68.6% | NA |
| Yue et al^67^ | Cohort | 21/1-11/2 2020 | China (Gansu) | 86 | IN | 44.2% | 41.0 (31.0-54.3) |
| Han et al^68^ | Cohort | 16/3-15/4 2020 | China (Beijing) | 53 | IN | 39.6% | NA |
| Huang et al^69^ | Cohort | 17/1-10/2 2020 | China (Hunan) | 37 | IN | 52.0% | 41.0 (31.0-51.0) |
| Zhai et al^70^ | Cohort | 15/3-30/4 2020 | China (Beijing) | 53 | IN | 39.6% | NA |
| Jin et al^71^ | Cohort | 17/1-8/2 2020 | China (Zhejiang) | 74 | IN | 50.0% | 46.1 |
| IIgin et al^72^ | Cohort | 1/4-30/4 2020 | Turkey | 91 | IN | 50.5% | 41.1 ± 15.4 |
| Li et al^73^ | Cohort | 20/1-10/6 2020 | China (Shanghai) | 475 | IN | 54.3% | 42.0 (32.0-58.0) |
| Wang et al^74^ | Cohort | 31/1-12/2 2020 | China (Shandong) | 26 | IN | 42.3% | 42.0 (34.0-53.0) |
| Qi et al^75^ | Cohort | 24/1-8/3 2020 | China (Hunan) | 137 | IN | 46.0% | 42.0 (35.0-54.0) |
| Yan et al^76^ | Cohort | 21/1-27/6 2020 | China (Hunan) | 218 | IN | 56.0% | 42.9 (32.0-52.3) |
| Dong et al^77^ | Case series | 19/1-14/2 2020 | China (Hubei) | 11 | IN | 45.5% | 43.0 (24.0-45.0) |
| Liu et al^78^ | Cohort | 22/1-15/3 2020 | China (Jilin) | 93 | IN | 58.1% | 43.0 (29.0-54.0) |
| Roozbeh et al^79^ | RCT + Randomised parallel group | 8/4-19/5 2020 | Iran | 55 | OUT | 47.0% | 43.0 (37.0-53.0) |
| Guo et al^80^ | Cohort | 21/1-15/3 2020 | China (Hunan) | 350 | IN | 49.4% | 43.0 (32.0-56.0) |
| Guan et al^81^ | Cohort | As of 30/6 2020 | China (Zhejiang) | 61 | IN | 62.3% | NA |
| Huang et al^82^ | Cohort | 18/1-26/2 2020 | China (Jiangsu) | 280 | IN | 52.1% | 43.0 (32.0-56.0) |
| Zhang et al^83^ | Case series | 23/1-5/2 2020 | China (Guangdong) | 12 | IN | 66.7% | 38.0 ± 16.3 |
| Cheng et al^84^ | Cohort | 12/2-8/3 2020 | China (Hubei) | 116 | IN | 57.8% | 44.0 (22.0-67.0) |
| Huang et al^85^ | Cohort | As 10/2 2020 | China (Jiangsu) | 202 | IN | 57.4% | 44.0 (33.0-54.0) |
| Hu et al^86^ | Cohort | 25/1-24/2 2020 | China (Zhejiang) | 16 | IN | 37.5% | 44.1 ± 16.5 |
| Mancilla-Galindo et al^87^ | Cohort | 24/2-14/9 2020 | Mexico | 136855 | MIX | 51.3% | 44.2 ± 16.8 |
| Zuo et al^88^ | Cohort | 21/1-16/3 2020 | China (Anhui) | 181 | IN | 55.2% | 44.3 ± 13.2 |
| Lin et al^89^ | Cohort | 17/1-15/2 2020 | China (Guangdong) | 95 | IN | 47.4% | 45.3 ± 18.3 |
| Liu et al^90^ | Cohort | 6/2-26/3 2020 | China (Hubei) | 68 | IN | 36.8% | 44.3 ± 16.4 |
| Chen et al^91^ | RCT + Randomised parallel group | 4/2-28/2 2020 | China (Hubei) | 62 | IN | 46.8% | 44.7 ± 15.3 |
| Demir et al^92^ | Cohort | 1/2-4/5 2020 | Turkey | 40 | MIX | 50.0% | 44.9 ± 14.8 |
| Chu et al^93^ | Cohort | 23/1-8/3 2020 | China (Zhejiang) | 48 | IN | 52.1% | 45.0 (24.0-59.0) |
| Zhang et al^94^ | Cohort | 13/1-16/2 2020 | China (Hubei) | 111 | IN | 41.4% | 45.0 (32.0-57.0) |
| Gao et al^95^ | Cohort | 20/1-15/3 2020 | China (Ningxia) | 73 | IN | 56.2% | 40.8 ± 17.0 |
| Wang et al^96^ | Cohort | 21/1-21/2 2020 | China (Hunan) | 169 | IN | 50.9% | 45.0 (34.5–55.0) |
| Lu et al^97^ | Cohort | 4/2-23/2 2020 | China (Jiangxi) | 47 | IN | 44.5% | 45.0 (34.0-50.0) |
| Cheng et al^98^ | RCT + Randomised parallel group | 18/2-10/4 2020 | China (Hubei) | 200 | IN | 56.0% | 45.0 (40.0-55.0) |
| Hong et al^99^ | Cohort | 21/1-7/2 2020 | China (Zhejiang) | 67 | IN | 53.7% | 45.0 ± 15.2 |
| Gao et al^100^ | Cohort | 21/1-18/2 2020 | China (Shaanxi) | 40 | IN | 47.5% | 41.0 ± 16.4 |
| Ma et al^101^ | Cohort | 26/1-8/3 2020 | China (Anhui) | 40 | IN | 50.0% | 43.4 ± 12.1 |
| Guo et al^102^ | Cohort | 21/1-19/2 2020 | China (Hunan) | 19 | IN | 50.6% | 45.2 |
| Shalimar et al^103^ | Cohort | 22/4-22/7 2020 | India | 24 | IN | 70.8% | 45.8 ± 12.7 |
| Luo et al^104^ | Cohort | 10/1-15/3 2020 | China (Jiangsu) | 625 | IN | 52.6% | 46.0 (32.0-57.0) |
| Jin et al^105^ | Cohort | 22/1-3/2 2020 | China (Jiangxi) | 45 | IN | 62.2% | 45.0 ± 16.4 |
| Tudoran et al^106^ | Cohort | 15/4-15/7 2020 | Romania | 91 | IN | 48.4% | 46.0 (40.0-50.0) |
| Zhang et al^107^ | Cohort | 23/1-21/3 2020 | China (Guangdong) | 257 | IN | 54.0% | 46.0 ± 17.0 |
| Wu et al^108^ | Cohort | 22/1-14/2 2020 | China (Jiangsu) | 80 | IN | 49.0% | 46.1 (30.7-61.5) |
| Ma et al^109^ | Cohort | 23/1-8/3 2020 | China (Hunan) | 450 | IN | 51.0% | 46.2 ± 15.1 |
| Zhang et al^110^ | Cohort | 28/1-1/3 2020 | China (Hubei) | 83 | IN | 52.0% | NA |
| Deng et al^111^ | Cohort | 17/1-20/3 2020 | China (Guangdong) | 83 | IN | 42.0% | NA |
| Wang et al^112^ | Cohort | 22/1-27/2 2020 | China (Fujian) | 199 | IN | 52.8% | 46.3 ± 16.4 |
| Zhang et al^113^ | Cohort | 20/1-30/3 2020 | China (Beijing) | 40 | IN | 52.5% | 46.4 ± 3.1 |
| Gupta et al^114^ | Cohort | 1/4-31/5 2020 | India | 85 | IN | 58.5% | NA |
| Wan et al^115^ | Cohort | 23/1-8/2 2020 | China (Chongqing) | 135 | IN | 53.3% | 47.0 (36.0‐55.0) |
| Plavunov et al^116^ | Case series | NA | Russia | 10 | OUT | NA | 47.0 (37.0-58.0) |
| Lai et al^117^ | Cohort | 11/1-10/2 2020 | China (Guangdong) | 330 | IN | 47.8% | 47.0 (33.0–60.0) |
| Guan et al^118^ | Cohort | As of 29/1 2020 | China (Multiregions) | 1099 | IN | 58.3% | 47.0 (35.0-58.0) |
| Abdulrahman et al^119^ | Cohort | 24/2-31/7 2020 | Bahrain | 1571 | IN | 59.4% | 47.1 ± 14.7 |
| Cai et al^120^ | Cohort | 11/1-6/3 2020 | China (Guangdong) | 298 | IN | 49.0% | 47.5 (33.0-61.0) |
| Zhang et al^121^ | Cohort | 1/1-29/2 2020 | China (Henan) | 172 | IN | 43.5% | 47.9 ± 18.3 |
| Shao et al^122^ | Cohort | 10/1-8/3 2020 | China (Hubei) | 18 | IN | 40.0% | 48.0 (33.0–63.0) |
| Saha et al^123^ | Cohort | 1/4-24/8 2020 | Bangladesh | 168 | IN | 79.8% | 48.0 (51.0-60.0) |
| Gao et al^124^ | Cohort | 12/1-29/2 2020 | China (Hubei) | 219 | IN | 50.2% | 48.0 (35.0 -60.0) |
| Jin et al^125^ | Cohort | 17/1-18/2 2020 | China (Guangdong) | 93 | IN | 44.1% | 48.0 (35.5-62.5) |
| Wu et al^126^ | Cohort | 23/1-2/3 2020 | China (Jiangsu) | 55 | IN | 51.0% | 45.2 ± 18.5 |
| Ye et al^127^ | Cohort | 1/1-31/3 2020 | China (Zhejiang) | 117 | IN | 55.6% | 48.2 ± 13.5 |
| Li et al^128^ | Cohort | 16/1-30/3 2020 | China (Hubei) | 96 | IN | 58.3% | NA |
| Zhang et al^129^ | Cohort | 22/1-28/2 2020 | China (Multiregions) | 194 | IN | 55.7% | 48.3 (33-56) |
| Xu et al^130^ | Cohort | 28/1-24/2 2020 | China (Shandong) | 35 | IN | 45.7% | 46.0 (35.0-54.0) |
| Lei et al^131^ | Cohort | 15/1-10/3 2020 | China (Guangdong) | 288 | IN | 45.5% | 48.5 (34.3-62.0) |
| Tian et al^132^ | Cohort | As of 10/3 2020 | China (Hubei) | 721 | IN | 48.0% | 48.5 ± 14.4 |
| Fatima et al^133^ | Cohort | 1/6-30/6 2020 | Pakistan | 100 | IN | NA | NA |
| Liu et al^134^ | Cohort | 20/1-10/2 2020 | China (Guangdong) | 278 | IN | 46.8% | 48.1 ± 17.0 |
| Liu et al^135^ | Cohort | 20/1-16/2 2020 | China (Jiangsu) | 90 | IN | 54.4% | 49.0 (33.0–60.0) |
| Chen et al^136^ | Cohort | 20/1-15/3 2020 | China (Guangdong) | 267 | IN | 45.3% | 49.0 (34.0-62.0) |
| Huang et al^137^ | Cohort | As of 2/1 2020 | China (Hubei) | 41 | IN | 73.2% | 49.0 (41.0-58.0) |
| Fan et al^138^ | Cohort | 20/1-31/1 2020 | China (Shanghai) | 148 | IN | 51.0% | 50.0 (36.0-64.0) |
| Zheng et al^139^ | Cohort | 5/2-9/3 2020 | China (Hubei) | 1320 | IN | 44.0% | 50.0 (40.0-57.0) |
| Alexandre et al^140^ | RCT + Randomised parallel group | 29/3-2/6 2020 | Brazil | 504 | IN | 60.1% | 50.0 ± 13.9 |
| Tanriverdi et al^141^ | Cross-sectional | 13/3-15/4 2020 | Turkey | 83 | IN | 72.2% | 50.0 ± 15.0 |
| Wang et al^142^ | Cohort | 1/1-31/3 2020 | China (Hubei) | 108 | IN | 66.6% | NA |
| Chen et al^143^ | Cohort | 16/1-21/2 2020 | China (Sichuan) | 98 | IN | 51.0% | NA |
| Xie et al^144^ | Cohort | 23/1-19/4 2020 | China (Hubei) | 25 | IN | 52.0% | 50.0 (33.0-86.0) |
| Li et al^145^ | Cohort | 21/1-10/3 2020 | China (Beijing) | 53 | IN | 41.5% | 50.2 ± 15.2 |
| Molla et al^146^ | Cohort | 21/5-10/6 2020 | Bangladesh | 193 | IN | 69.4% | 50.4 ± 14.1 |
| Sekhawati et al^147^ | RCT + Randomised parallel group | 24/4-8/5 2020 | Iran | 111 | IN | 46.0% | NA |
| Yuz et al^148^ | Cohort | 15/3-31/5 2020 | Turkey | 479 | IN | 50.5% | 50.7 ± 19.3 |
| Ji et al^149^ | Cohort | 2/1-28/1 2020 | China (Hubei) | 101 | IN | 48.0% | 51.0 (37.0-61.0) |
| Wu et al^150^ | Cohort | 25/12/2019-26/1/2020 | China (Hubei) | 201 | IN | 63.7% | 51.0 (43.0-60.0) |
| Borba et al^151^ | RCT + Randomised parallel group | 23/3-15/4 2020 | Brazil | 81 | IN | 74.0% | 51.1 ± 13.9 |
| Zhang et al^152^ | Cohort | 1/1-31/3 2020 | China (Hubei) | 90 | IN | 54.4% | 51.8 ± 17.6 |
| Yan et al^153^ | Cohort | 20/1-24/3 2020 | China (Hubei) | 116 | IN | 0.0% | NA |
| Martinez-Guerra et al^154^ | Cohort | 20/3-10/6 2020 | Mexico | 794 | IN | 61.6% | 52.0 (43.0-62.0) |
| Yao et al^155^ | Cohort | 30/1-11/2 2020 | China (Hubei) | 108 | IN | 40.0% | 52.0 (37.0-58.0) |
| Meng et al^156^ | Cohort | 27/1-10/3 2020 | China (Hubei) | 108 | IN | 38.0% | NA |
| Wang et al^157^ | Cohort | 23/1-3/3 2020 | China (Hubei) | 40 | IN | 37.5% | 52.3 ± 8.3 |
| Chen et al^158^ | Cohort | 24/1-29/2 2020 | China (Hubei) | 118 | IN | NA | NA |
| Feng et al^159^ | Cohort | 1/1-15/2 2020 | China (Multiregions) | 476 | IN | 56.9% | 53.0 (40.0-64.0) |
| Xiong et al^160^ | Cohort | 1/1-29/2 2020 | China (Hubei) | 123 | IN | 49.6% | NA |
| Lai et al^161^ | Cohort | 23/1-22/4 2020 | China (Guangdong) | 125 | IN | NA | NA |
| Wang et al^162^ | Cohort | 17/1-27/3 2020 | China (Hubei) | 356 | IN | 50.8% | 53.0 (40.0-65.0) |
| Galan et al^163^ | RCT + Randomised parallel group | 1/4-31/12 2020 | Brazil | 168 | IN | 56.5% | 53.4 ± 15.6 |
| Liu et al^164^ | Cohort | 1/1-29/2 2020 | China (Shanghai) | 200 | IN | 53.0% | 54.0 (37.0–65.0) |
| Pan et al^165^ | Cross-sectional | 18/1-28/2 2020 | China (Hubei) | 204 | IN | 52.5% | 52.9 ± 16.0 |
| Peng et al^166^ | Cohort | As of 29/1 2020 | China (Hubei) | 75 | IN | 58.7% | 6.1 ± 4.8 |
| Yi et al^167^ | Cohort | 19/1-19/2 2020 | China (Zhejiang) | 100 | IN | 63.0% | 54.0 (42.0–64.0) |
| Li et al^168^ | RCT + Randomised parallel group | 10/2-5/4 2020 | China (Multiregions) | 94 | IN | 46.8% | 54.0 (39.8–63.3) |
| Xie et al^169^ | Cohort | 1/1-15/2 2020 | China (Shanghai) | 21 | IN | 61.9% | 54.0 ± 17.9 |
| Shi et al^170^ | Cohort | 30/1-23/3 2020 | China (Hubei) | 60 | IN | 66.7% | 54.5 (45.0 - 64.8 ) |
| Peymani et al^171^ | Cohort | 1/3-30/5 2020 | Iran | 150 | IN | 42.0% | 54.7 ± 4.4 |
| Lin et al^172^ | Cohort | 24/1-10/3 2020 | China (Hubei) | 94 | IN | 49.0% | 53.3 ± 15.3 |
| Tong et al^173^ | Cohort | 1/1-29/2 2020 | China (Hubei) | 115 | IN | 53.9% | 54.9 ± 15.1 |
| Akbariqomi et al^174^ | Cohort | 26/2-26/3 2020 | Iran | 595 | IN | 67.4% | 55.0 (45.0-63.0) |
| Wu et al^175^ | Cohort | 25/1-18/4 2020 | China (Hubei) | 157 | IN | 40.5% | NA |
| Malekzadeh et al^176^ | RCT + Randomised parallel group | 15/3-22/6 2020 | Iran | 126 | IN | 63.5% | 55.0 (46.0–63.0) |
| Wang et al^177^ | Cohort | 16/1-29/1 2020 | China (Hubei) | 69 | IN | 46.0% | 41.0 (35.0-62.0) |
| Cheng et al^178^ | Cohort | 1/1-20/3 2020 | China (Hubei) | 456 | IN | 46.2% | 55.0 ± 18.6 |
| de Melo et al^179^ | Cohort | 30/4-26/5 2020 | Brazil | 181 | IN | 39.2% | 55.3 ± 2.1 |
| Chen et al^180^ | Cohort | 1/1-20/1 2020 | China (Hubei) | 99 | IN | 67.7% | 55.5 ± 13.1 |
| Najafi et al^181^ | Cohort | 20/2-19/3 2020 | Iran | 978 | IN | 67.0% | 55.6 ± 15.6 |
| Zhang et al^182^ | Cohort | 17/1-18/5 2020 | China (Hubei) | 35 | IN | 0.0% | 56.0 (42.0-62.0) |
| Wang et al^183^ | Cohort | 1/1-3/2 2020 | China (Hubei) | 138 | IN | 54.3% | 56.0 (42.0-68.0) |
| Xiao et al^184^ | RCT + Randomised parallel group | 5/2-10/2 2020 | China (Hubei) | 182 | IN | 56.5% | NA |
| Xu et al^185^ | Cohort | 20/1-10/3 2020 | China (Guangdong) | 187 | IN | 43.8% | NA |
| Yang et al^186^ | Cohort | 30/1-26/2 2020 | China (Hubei) | 200 | IN | 49.5% | 55.0 ± 17.1 |
| Zhao et al^187^ | Cohort | 27/1-1/4 2020 | China (Henan) | 29 | IN | 48.3% | 56.0 (31.5-66.0) |
| Pei et al^188^ | Cohort | 28/1-9/2 2020 | China (Hubei) | 198 | IN | 54.7% | 56.3 ± 13.4 |
| Chen et al^189^ | Cohort | 25/12/2019-3/3/2020 | China (Hubei) | 1105 | IN | 54.6% | 56.3 ± 14.5 |
| Hu et al^190^ | Cohort | 18/1-11/2 2020 | China (Hubei) | 14 | IN | 57.0% | 56.7 ± 15.3 |
| Xu et al^191^ | Cohort | 14/1-20/2 2020 | China (Guangdong) | 45 | IN | 64.4% | 56.7 ± 15.4 |
| Bosevski et al^192^ | Case series | NA | North Macedonia | 17 | IN | NA | 57.0 ± 14.0 |
| Shu et al^193^ | Cohort | 15/1-2/3 2020 | China (Hubei) | 293 | IN | 46.0% | 57.1 ± 15.6 |
| Namendys-Silva et al^194^ | Cohort | 1/4-30/4 2020 | Mexico | 164 | IN | 70.0% | 57.3 ± 13.7 |
| Sun et al^195^ | Cohort | 1/1-30/4 2020 | China (Hubei) | 30 | IN | 50.0% | 57.5 (50.8-66.8) |
| Solaymani-Dodaran et al^196^ | RCT + Randomised parallel group | 2/4-3/8 2020 | Iran | 373 | IN | 55.0% | 57.6 ± 17.3 |
| Chen et al^197^ | Cohort | 25/1-9/3 2020 | China (Hubei) | 271 | IN | 53.5% | 57.7 ± 14.3 |
| Sadeghi et al^198^ | RCT + Randomised parallel group | 26/3-26/4 2020 | Iran | 66 | IN | 52.0% | 58.0 (43.0-69.0) |
| Cao et al^199^ | RCT + Randomised parallel group | 18/1-3/2 2020 | China (Hubei) | 191 | IN | 60.3% | 58.0 (49.0-68.0) |
| Zhang et al^200^ | Cohort | 29/1-23/3 2020 | China (Hubei) | 97 | IN | 44.3% | 58.0 (42.5-67.0) |
| Wang et al^201^ | Cohort | 17/1-25/2 2020 | China (Hubei) | 55 | IN | 52.7% | 58.0 (43.5-66.5) |
| Peng et al^202^ | Cohort | 11/1-8/3 2020 | China (Hubei) | 205 | IN | 47.8% | 58.4 ± 13.5 |
| Davoudi-Monfared et al^203^ | RCT + Randomised parallel group | 29/2-3/4 2020 | Iran | 81 | IN | 54.3% | 58.8 (48.6-68.6) |
| Cheng et al^204^ | Cohort | NA | China (Hubei) | 290 | IN | 43.5% | 54.8 ± 4.5 |
| Li et al^205^ | Cohort | 1/2-29/2 2020 | China (Hubei) | 132 | IN | 56.9% | 58.8 ± 12.9 |
| Chen et al^206^ | Cohort | 22/1-25/3 2020 | China (Hebei) | 51 | IN | 47.0% | 58.9 ± 13.7 |
| Li et al^207^ | Cohort | 20/1-4/4 2020 | China (Hubei) | 1859 | IN | 50.2% | 59.0 (45.0-68.0) |
| Tang et al^208^ | Cohort | 20/1-10/3 2020 | China (Hubei) | 120 | IN | 45.0% | 59.0 (47.0-68.0) |
| Marcolino et al^209^ | Cohort | 1/3-19/9 2020 | Brazil | 2054 | IN | 52.6% | 59.0 (47.0-71.0) |
| Li et al^210^ | Cohort | 15/2-30/3 2020 | China (Hubei) | 1643 | IN | 47.6% | 59.0 (49.0-68.0) |
| Zhou et al^211^ | Cohort | 11/1-18/2 2020 | China (Hubei) | 220 | IN | 47.3% | 59.5 (47.0-69.0) |
| Molaei et al^212^ | Cohort | 8/2-28/3 2020 | Iran | 10 | IN | 80.0% | 59.6 ± 7.7 |
| Du et al^213^ | Cohort | 28/1-28/2 2020 | China (Hubei) | 182 | IN | 66.0% | 6 (0.0-15.0) |
| Yang et al^214^ | Cohort | 28/1-12/2 2020 | China (Hubei) | 136 | IN | 48.5% | 56.0 (44.0-64.0) |
| Lima-Setta et al^215^ | Cohort | 1/3-31/7 2020 | Brazil | 56 | IN | 70.0% | 6.2 (2.4-10.3) |
| Ramadan et al^216^ | Cohort | 3/5-30/6 2020 | Egypt | 260 | IN | 55.4% | NA |
| Lian et al^217^ | Cohort | 2/2-20/3 2020 | China (Hubei) | 81 | IN | 56.0% | 60.0 (49.0-66.0) |
| Zhao et al^218^ | Cohort | 29/1-19/2 2020 | China (Hubei) | 413 | IN | 51.0% | 60.3 ± 12.7 |
| Ding et al^219^ | Cohort | 29/1-15/3 2020 | China (Hubei) | 115 | IN | 53.0% | 60.5 ± 12.2 |
| Wu et al^220^ | Cohort | 20/1-24/2 2020 | China (Hubei) | 382 | IN | 61.0% | 60.7 ± 14.1 |
| Cen et al^221^ | Cohort | As of 10/2 2020 | China (Hubei) | 1007 | IN | 49.0% | 61.0 (49.0-68.0) |
| Wang et al^222^ | Cohort | 19/1-19/2 2020 | China (Hubei) | 260 | IN | 50.8% | 61.0 (42.0-73.0) |
| Liu et al^223^ | Cohort | 26/1-18/3 2020 | China (Hubei) | 1123 | IN | 50.0% | 61.0 (50.0-69.0) |
| Hu et al^224^ | Cohort | 1/1-18/3 2020 | China (Guangdong) | 64 | IN | 62.5% | 61.0 (49.3-67.5) |
| Ussaid et al^225^ | Cohort | 15/4-31/8 2020 | Pakistan | 47 | IN | 61.7% | 61.5 ± 13.4 |
| Davidescu et al^226^ | Cohort | 1/5-23/8 2020 | Romania | 22 | IN | 45.5% | 61.7 ± 14.5 |
| Wu et al^227^ | Cohort | 30/1-10/3 2020 | China (Hubei) | 101 | IN | 54.0% | 62.0 (49.0-72.0) |
| Chen et al^228^ | Cohort | 18/1-27/3 2020 | China (Hubei) | 3309 | IN | 49.6% | 62.0 (49.0-69.0) |
| Wang et al^229^ | Cohort | 9/3-17/3 2020 | China (Hubei) | 116 | IN | 53.4% | 62.0 (55.0-69.0) |
| Zhang et al^230^ | Case control | 20/1-20/3 2020 | China (Beijing) | 216 | IN | 50.0% | NA |
| Cheng et al^231^ | Cohort | 18/1-28/2 2020 | China (Hubei) | 1392 | IN | 51.0% | 63.0 (50.0-71.0) |
| Shi et al^232^ | Cohort | 1/1-23/2 2020 | China (Hubei) | 671 | IN | 48.0% | 63.0 (50.0-72.0) |
| Yang et al^233^ | Cohort | 13/1-18/3 2020 | China (Hubei) | 205 | IN | 47.0% | 63.0 (56.0-70.0) |
| Furtado et al^234^ | RCT + Randomised parallel group | 28/3-19/5 2020 | Brazil | 397 | IN | 66.0% | NA |
| Cao et al^235^ | RCT + Randomised parallel group | 9/2-28/2 2020 | China (Hubei) | 41 | IN | 58.5% | 63.0 (58.0-68.0) |
| Peng et al^236^ | Cohort | 1/2-25/3 2020 | China (Hubei) | 49.00 | IN | 65.3% | 63.0 (53.0-73.0) |
| Song et al^237^ | Cohort | 1/2-6/3 2020 | China (Hubei) | 961 | IN | 52.0% | 63.0 (49.0-70.0) |
| Ma et al^238^ | Cohort | 31/1-29/2 2020 | China (Hubei) | 109 | IN | 46.8% | 63.0 (50.3-71.8) |
| Salehi et al^239^ | Cross-sectional | 1/3-30/4 2020 | Iran | 53 | IN | 43.4% | 63.1 ± 16.4 |
| Agarwal et al^240^ | RCT + Randomised parallel group | 22/4-14/7 2020 | India | 235 | IN | 75.0% | NA |
| Lian et al^241^ | Cohort | 17/1-31/1 2020 | China (Zhejiang) | 465 | IN | 52.3% | NA |
| Tian et al^242^ | Case control | 18/1-18/2 2020 | China (Hubei) | 90 | IN | 53.0% | 64.0 (56.0-70.0) |
| Yu et al^243^ | Cross-sectional | 8/2-27/2 2020 | China (Hubei) | 226 | IN | 61.5% | 64.0 (57.0-70.0) |
| Feng et al^244^ | Cohort | 23/1-22/2 2020 | China (Hubei) | 114 | IN | 62.3% | 64.0 ± 13.4 |
| Huang et al^245^ | Cohort | 29/1-8/3 2020 | China (Hubei) | 17 | IN | 17.6% | 64.0 (60.5-71.5) |
| Yang et al^246^ | Cohort | 24/1-8/3 2020 | China (Hubei) | 358 | IN | NA | NA |
| Zhang et al^247^ | Cohort | 29/1-12/2 2020 | China (Hubei) | 258 | IN | 53.5% | 64.0 (56.0-70.0) |
| Liang et al^248^ | Cohort | 1/1-29/2 2020 | China (Hubei) | 542 | IN | NA | NA |
| Chen et al^249^ | Cohort | 3/1-9/4 2020 | China (Hubei) | 681 | IN | 53.2% | 65.0 (54.0-72.0) |
| Hui et al^250^ | Cohort | 28/1-10/3 2020 | China (Hubei) | 167 | IN | 65.3% | 65.0 (56.0-72.0) |
| Du et al^251^ | Cohort | 9/1-15/2 2020 | China (Hubei) | 85 | IN | 73.0% | 65.8 ± 14.2 |
| Wang et al^252^ | Cohort | 1/1-31/3 2020 | China (Hubei) | 315 | IN | 45.0% | NA |
| Li et al^253^ | Cohort | 25/1-26/2 2020 | China (Hubei) | 74 | IN | 59.5% | 66.0 (55.0-72.0) |
| Yu et al^254^ | Cohort | 1/2-26/3 2020 | China (Hubei) | 689 | IN | 52.8% | 66.0 (57.0–73.0) |
| Zheng et al^255^ | Cohort | 22/1-5/3 2020 | China (Zhejiang) | 34 | IN | 67.6% | 66.0 (58.0-76.0) |
| Wang et al^256^ | Cohort | 29/1-20/3 2020 | China (Hubei) | 156 | IN | 48.7% | 66.0 (46.3–73.0) |
| Zhou et al^257^ | Cohort | 28/1-2/3 2020 | China (Hubei) | 21 | IN | 62.0% | 66.1 ± 13.9 |
| Li et al^258^ | Cohort | 27/1-17/3 2020 | China (Hubei) | 102 | IN | 66.7% | 66.2 (30-93) |
| Wang et al^259^ | Cohort | 1/2-18/3 2020 | China (Zhejiang) | 22 | IN | 77.3% | 66.3 ± 15.1 |
| Ji et al^260^ | Cohort | 24/2-4/5 2020 | China (Multiregions) | 27 | IN | 37.0% | 66.4 ± 12.1 |
| Liu et al^261^ | Cohort | 8/2-15/4 2020 | China (Hubei) | 934 | IN | 48.6% | NA |
| Xia et al^262^ | Cohort | 5/2-20/3 2020 | China (Hubei) | 81 | IN | 66.7% | 66.6 ± 11.4 |
| Wang et al^263^ | Cohort | 17/2-18/3 2020 | China (Hubei) | 210 | IN | 47.6% | 67.0 (59.8-74.0) |
| Sharifipour et al^264^ | Cohort | NA | Iran | 19 | IN | 58.0% | 67.1 ± 14.6 |
| Mannan et al^265^ | Cross-sectional | 1/4-30/6 2020 | Bangladesh | 1021 | IN | 76.1% | NA |
| Wu et al^266^ | Cohort | 11/12/2019-20/2/2020 | China (Multiregions) | 1048 | MIX | 83.0% | NA |
| Wang et al^267^ | Cohort | 2/2-1/3 2020 | China (Hubei) | 59 | IN | 64.4% | 67.4 ± 11.3 |
| Li et al^268^ | Cohort | 31/1-20/2 2020 | China (Hubei) | 204 | IN | 49.0% | 68.0 (64.0-75.0) |
| Temel et al^269^ | Cohort | 9/3-22/4 2020 | Turkey | 47 | IN | 66.0% | 68.0 ± 13.0 |
| Li et al^270^ | Case series | 1/2-31/3 2020 | China (Hubei) | 30 | IN | 66.7% | 68.5 (61.2-77.8) |
| Yu et al^271^ | Cohort | 1/2-4/4 2020 | China (Hubei) | 550 | IN | 62.5% | 69.0 (59.0-77.0) |
| Dai et al^272^ | Cohort | 29/1-25/2 2020 | China (Hubei) | 492 | IN | 46.0% | 69.0 (59.0-78.0) |
| Chen et al^273^ | Cohort | As of 31/1 2020 | China (Multiregions) | 1590 | IN | 2.0% | NA |
| Du et al^274^ | Cohort | As of 24/2 2020 | China (Hubei) | 109 | IN | 67.9% | 70.7 ± 10.9 |
| Yao et al^275^ | Cohort | 26/1-28/2 2020 | China (Hubei) | 83 | IN | 64.0% | 71.8 ± 13.2 |
| Mei et al^276^ | Cohort | 1/1-31/3 2020 | China (Hubei) | 223 | IN | 50.2% | 72.0 (68.0-77.5) |
| Li et al^277^ | Cohort | 1/2-31/3 2020 | China (Gansu) | 2464 | IN | 49.0% | NA |
| Zhu et al^278^ | Cohort | 7/1-4/3 2020 | China (Hubei) | 37 | IN | 56.8% | 76.3 ± 19.4 |
| Wong et al^279^ | Cohort | 21/1-6/12 2020 for Hongkong; and 1/1-27/2 2020 for China Anhui | China (Multiregions) | 5419 | IN | 48.2% | NA |
| Yala et al^280^ | Cohort | 11/3-23/5 2020 | Turkey | 77 | IN | 45.5% | 8.0 (2.0-13.0) |
| Zhu et al^281^ | Case series | 24/1-22/2 2020 | China (Jiangsu) | 10 | IN | 50.0% | 9.0 (5.0-11.5) |
| Cao et al^282^ | Cohort | 1/12/2019-3/3/2020 | China (Hunan) | 32 | IN | 56.3% | 43.8 ± 13.7 |
| Adriana et al^283^ | Cohort | NA | Peru, Mexico, Colombia, Costa Rico | 921 | IN | NA | NA |
| Tizkam et al^284^ | Cohort | 1/1-31/5 2020 | Iraq | 1324 | IN | NA | NA |

^*^ Abbreviations of settings: IN – inpatients; OUT – outpatients; MIX – mixed patients

^#^ Age was presented either Median (IQR) or Mean ± SD.

NA – data not avaiable

**References**

1. Prata-Barbosa A, Lima-Setta F, Santos GRd, et al. Pediatric patients with COVID-19 admitted to intensive care units in Brazil: a prospective multicenter study. *Jornal de Pediatria* 2020; **96**(5): 582-92.

2. Wu Q, Xing Y, Shi L, et al. Coinfection and Other Clinical Characteristics of COVID-19 in Children. *Pediatrics* 2020; **146**(1): e20200961.

3. Jing Y, Chaoying X, Chengying Y. 新型冠状病毒肺炎普通型病例40例临床研究. *实用心脑肺血管病杂志* 2020; **28**(2): 1-4.

4. Chen Z, Tong L, Zhou Y, et al. Childhood COVID-19: a multicentre retrospective study. *Clin Microbiol Infect* 2020; **26**(9): 1260.e1-.e4.

5. Nan Y, Jianli W, Ling F, et al. 妊娠晚期合并新冠肺炎的围生期结局. *现代妇产科进展* 2020; **3**: 167-9.

6. Jothimani D, Kailasam E, Danielraj S, et al. COVID-19: Poor outcomes in patients with zinc deficiency. *International Journal of Infectious Diseases* 2020; **100**: 343-9.

7. Gu X, Li X, An X, et al. Elevated serum aspartate aminotransferase level identifies patients with coronavirus disease 2019 and predicts the length of hospital stay. *J Clin Lab Anal* 2020; **34**(7): e23391.

8. Cao J, Tu W-J, Cheng W, et al. Clinical Features and Short-term Outcomes of 102 Patients with Coronavirus Disease 2019 in Wuhan, China. *Clinical Infectious Diseases* 2020; **71**(15): 748-55.

9. Yalin L, Nanbing S, Wei S, Baogui WD, Li. 普通型与重型/危重型新型冠状病毒肺炎患者临床特征的对比研究. *实用心脑肺血管病杂志* 2020; **28**(3): 14-9.

10. Feng G, Huang W-Q, Liu M-L, et al. Clinical Features of COVID-19 Patients in Xiaogan City. *SN Comprehensive Clinical Medicine* 2020; **2**(10): 1717-23.

11. Zhang X, Wang H, Wang Y, et al. Epidemiological and clinical based study on four passages of COVID-19 patients: intervention at asymptomatic period contributes to early recovery. *BMC Infectious Diseases* 2020; **20**(1): 855.

12. Fan L, Liu H, Li N, et al. Medical treatment of 55 patients with COVID-19 from seven cities in northeast China who fully recovered: A single-center, retrospective, observational study. *Medicine (Baltimore)* 2021; **100**(2): e23923.

13. Ruibin L, Wenju W, Xin L. 连花清瘟颗粒联合西药常规疗法治疗新型 冠状病毒肺炎疑似病例63例临床观察. *中医杂志* 2020; **61**(8): 655-9.

14. Chowdhary A, Tarai B, Singh A, Sharma A. Multidrug-Resistant Candida auris Infections in Critically Ill Coronavirus Disease Patients, India, April-July 2020. *Emerg Infect Dis* 2020; **26**(11): 2694-6.

15. Tang W, Cao Z, Han M, et al. Hydroxychloroquine in patients with mainly mild to moderate coronavirus disease 2019: open label, randomised controlled trial. *BMJ* 2020; **369**: m1849.

16. Gao Y, Ma X, Bi J, et al. Epidemiological and clinical differences of coronavirus disease 2019 patients with distinct viral exposure history. *Virulence* 2020; **11**(1): 1015-23.

17. Qiu Z, Dai W, Syeda MZ, et al. Clinical features of 64 patients (outside Hubei) with COVID-19 in Wenzhou, China. *J Thorac Dis* 2020; **12**(10): 6127-31.

18. Bastug A, Bodur H, Erdogan S, et al. Clinical and laboratory features of COVID-19: Predictors of severe prognosis. *International Immunopharmacology* 2020; **88**: 106950.

19. Wang Y, Liu S, Liu H, et al. SARS-CoV-2 infection of the liver directly contributes to hepatic impairment in patients with COVID-19. *J Hepatol* 2020; **73**(4): 807-16.

20. Soltani J, Sedighi I, Shalchi Z, Sami G, Moradveisi B, Nahidi S. Pediatric coronavirus disease 2019 (COVID-19): An insight from west of Iran. *North Clin Istanb* 2020; **7**(3): 284-91.

21. Ding R, Yang Z, Huang D, et al. Identification of parameters in routine blood and coagulation tests related to the severity of COVID-19. *Int J Med Sci* 2021; **18**(5): 1207-15.

22. Zengli L, Xiuhui L, Chunyan G, et al. Effect of Jinhua Qinggan granules on novel coronavirus pneumonia in patients. *Journal of Traditional Chinese Medicine* 2020; **40**(3): 467-72.

23. Deng W, Guang T-w, Yang M, et al. Positive results for patients with COVID-19 discharged form hospital in Chongqing, China. *BMC Infectious Diseases* 2020; **20**(1): 429.

24. Hu W, Lv X, Li C, et al. Disorders of sodium balance and its clinical implications in COVID-19 patients: a multicenter retrospective study. *Intern Emerg Med* 2021; **16**(4): 853-62.

25. Yusheng Z, Chunting W, Wei Z, Ximing W, Liang D. 山东省新型冠状病毒肺炎患者537例临床特征与救治效果. *山东大学学报* 2020; **58**(3): 44-51.

26. Bustos-Cordova E, Castillo-Garcia D, Ceron-Rodriguez M, Soler-Quinones N. Clinical Spectrum of COVID-19 in a Mexican Pediatric Population. *Indian Pediatr* 2021; **58**(2): 126-8.

27. Yuan H, Liu J, Gao Z, Hu F. Clinical Features and Outcomes of Acute Kidney Injury in Patients Infected with COVID-19 in Xiangyang, China. *Blood Purif* 2021; **50**(4-5): 513-9.

28. Abad CL, Lansang MAD, Cordero CP, et al. Early experience with COVID-19 patients in a private tertiary hospital in the Philippines: Implications on surge capacity, healthcare systems response, and clinical care. *Clinical Epidemiology and Global Health* 2021; **10**: 100695.

29. Li J, Li M, Zheng S, et al. Plasma albumin levels predict risk for nonsurvivors in critically ill patients with COVID-19. *Biomark Med* 2020; **14**(10): 827-37.

30. Camelo-Filho AE, Silva AMS, Estephan EP, et al. Myasthenia Gravis and COVID-19: Clinical Characteristics and Outcomes. *Frontiers in Neurology* 2020; **11**.

31. Chen L, Wang G, Long X, et al. Dynamics of Blood Viral Load Is Strongly Associated with Clinical Outcomes in Coronavirus Disease 2019 (COVID-19) Patients: A Prospective Cohort Study. *The Journal of Molecular Diagnostics* 2021; **23**(1): 10-8.

32. Zhang SS, Dong L, Wang GM, et al. Progressive liver injury and increased mortality risk in COVID-19 patients: A retrospective cohort study in China. *World J Gastroenterol* 2021; **27**(9): 835-53.

33. Wang Y, Zhang D, Du G, et al. Remdesivir in adults with severe COVID-19: a randomised, double-blind, placebo-controlled, multicentre trial. *The Lancet* 2020; **395**(10236): 1569-78.

34. Cheng Y, Luo R, Wang K, et al. Kidney disease is associated with in-hospital death of patients with COVID-19. *Kidney International* 2020; **97**(5): 829-38.

35. Escobar GM, Javier; Taype, Waldo; Ayala, Ricardo; Amado, Jose. Clinical epidemiological characteristics of patients deceased by COVID-19 in a national hospital in Lima, Peru. *Revista de la Facultad de Medicina Humana* 2020; **20**(2): 180-5.

36. Dang J-z, Zhu G-y, Yang Y-j, Zheng F. Clinical characteristics of coronavirus disease 2019 in patients aged 80 years and older. *Journal of Integrative Medicine* 2020; **18**(5): 395-400.

37. Sun L, Shen L, Fan J, et al. Clinical features of patients with coronavirus disease 2019 from a designated hospital in Beijing, China. *J Med Virol* 2020; **92**(10): 2055-66.

38. Fu H-Y, Luo Y, Gao J-P, et al. Effects of Short-Term Low-Dose Glucocorticoids for Patients with Mild COVID-19. *BioMed Research International* 2020; **2020**: 2854186.

39. Zhang Y, Xiao L-s, Li P, et al. Clinical Characteristics of Patients With Progressive and Non-progressive Coronavirus Disease 2019: Evidence From 365 Hospitalised Patients in Honghu and Nanchang, China. *Frontiers in Medicine* 2020; **7**.

40. Zhaosong D, Hua X, Maochang L. 武汉市新型冠状病毒感染患儿用药情况的回顾性分析. *中国当代儿科杂志* 2021; **23**(1): 61-6.

41. Bo F, Shihong J, Zhengxing L, Zhengrong M. 河南新县新型冠状病毒肺炎临床特点及诊断困难患者的诊疗策略. *中国医学工程* 2021; **29**(2): 6-12.

42. Zhang J, Cao F, Wu S-K, et al. Clinical characteristics of 31 hemodialysis patients with 2019 novel coronavirus: a retrospective study. *Renal Failure* 2020; **42**(1): 726-32.

43. Teich VD, Klajner S, Almeida FAS, et al. Epidemiologic and clinical features of patients with COVID-19 in Brazil. *Einstein (Sao Paulo)* 2020; **18**: eAO6022.

44. Abrishami A, Samavat S, Behnam B, Arab-Ahmadi M, Nafar M, Sanei Taheri M. Clinical Course, Imaging Features, and Outcomes of COVID-19 in Kidney Transplant Recipients. *Eur Urol* 2020; **78**(2): 281-6.

45. Rabha AC, Oliveira Junior FI, Oliveira TA, et al. CLINICAL MANIFESTATIONS OF CHILDREN AND ADOLESCENTS WITH COVID-19: REPORT OF THE FIRST 115 CASES FROM SABARÁ HOSPITAL INFANTIL. *Rev Paul Pediatr* 2020; **39**: e2020305.

46. Soh TV, Dzawani M, Noorlina N, Nik F, Norazmi A. Clinical characteristics of severe acute respiratory syndrome Coronavirus 2 (SARS-CoV2) patients in Hospital Tengku Ampuan Afzan. *Med J Malaysia* 2020; **75**(5): 479-84.

47. Merza MA, Haleem Al Mezori AA, Mohammed HM, Abdulah DM. COVID-19 outbreak in Iraqi Kurdistan: The first report characterizing epidemiological, clinical, laboratory, and radiological findings of the disease. *Diabetes & Metabolic Syndrome: Clinical Research & Reviews* 2020; **14**(4): 547-54.

48. Zeng Y, Lin L, Yan Q, et al. Update on clinical outcomes of women with COVID-19 during pregnancy. *International Journal of Gynecology & Obstetrics* 2020; **150**(2): 264-6.

49. Liu D, Li L, Wu X, et al. Pregnancy and Perinatal Outcomes of Women With Coronavirus Disease (COVID-19) Pneumonia: A Preliminary Analysis. *American Journal of Roentgenology* 2020; **215**(1): 127-32.

50. Xu S, Shao F, Bao B, et al. Clinical Manifestation and Neonatal Outcomes of Pregnant Patients With Coronavirus Disease 2019 Pneumonia in Wuhan, China. *Open Forum Infectious Diseases* 2020; **7**(7): ofaa283.

51. Hengyi Y, Xiuhua R, Xingxing Q, Qin Z, Dong L. 阿比多尔、清肺排毒汤、连花清瘟胶囊、金叶败毒颗粒对某方舱医院轻型/普通型新冠肺炎患者疗效的回顾性研究. *中药药理与临床* 2020; **36**(6): 2-6.

52. Wei L, Gao X, Chen S, et al. Clinical Characteristics and Outcomes of Childbearing-Age Women With COVID-19 in Wuhan: Retrospective, Single-Center Study. *J Med Internet Res* 2020; **22**(8): e19642.

53. Wei X-S, Wang X-R, Zhang J-C, et al. A cluster of health care workers with COVID-19 pneumonia caused by SARS-CoV-2. *Journal of Microbiology, Immunology and Infection* 2021; **54**(1): 54-60.

54. Kosugi EM, Lavinsky J, Romano FR, et al. Incomplete and late recovery of sudden olfactory dysfunction in COVID-19. *Braz J Otorhinolaryngol* 2020; **86**(4): 490-6.

55. Wang X, Jiang X, Huang Q, et al. Risk factors of SARS-CoV-2 infection in healthcare workers: a retrospective study of a nosocomial outbreak. *Sleep Medicine: X* 2020; **2**: 100028.

56. Ye XT, Luo YL, Xia SC, et al. Clinical efficacy of lopinavir/ritonavir in the treatment of Coronavirus disease 2019. *Eur Rev Med Pharmacol Sci* 2020; **24**(6): 3390-6.

57. Huang C, Fei L, Li W, et al. Efficacy evaluation of intravenous immunoglobulin in non-severe patients with COVID-19: A retrospective cohort study based on propensity score matching. *International Journal of Infectious Diseases* 2021; **105**: 525-31.

58. Liu C, Dun Y, Liu P, et al. Associations of medications used during hospitalization and immunological changes in patients with COVID-19 during 3-month follow-up. *International Immunopharmacology* 2020; **89**: 107121.

59. Ye G, Pan Z, Pan Y, et al. Clinical characteristics of severe acute respiratory syndrome coronavirus 2 reactivation. *Journal of Infection* 2020; **80**(5): e14-e7.

60. Nguyen TT, Pham TN, Van TD, et al. Genetic diversity of SARS-CoV-2 and clinical, epidemiological characteristics of COVID-19 patients in Hanoi, Vietnam. *PLoS One* 2020; **15**(11): e0242537.

61. Fei X, Ming C, Wei A, et al. 武汉某院早期COVID-19患者临床特征与诊疗数据的回顾性研究. *中国医院药学杂志* 2021; **41**(3): 287-93.

62. Wang R, Pan M, Zhang X, et al. Epidemiological and clinical features of 125 Hospitalized Patients with COVID-19 in Fuyang, Anhui, China. *International Journal of Infectious Diseases* 2020; **95**: 421-8.

63. Zhong Q, Liu YY, Luo Q, et al. Spinal anaesthesia for patients with coronavirus disease 2019 and possible transmission rates in anaesthetists: retrospective, single-centre, observational cohort study. *Br J Anaesth* 2020; **124**(6): 670-5.

64. Ilic I, Zdravkovic M, Timcic S, Stojanovic DU, Bojic M, Loncar G. Pneumonia in medical professionals during COVID-19 outbreak in cardiovascular hospital. *International Journal of Infectious Diseases* 2021; **103**: 188-93.

65. Chufen L, Xuebin L, Yongneng J, et al. 新型冠状病毒肺炎轻症患者发病早期临床诊治与发热的相关性. *海南医学院学报* 2020; **41**(3): 287-93.

66. Huang H, Li H, Chen S, et al. Prevalence and Characteristics of Hypoxic Hepatitis in COVID-19 Patients in the Intensive Care Unit: A First Retrospective Study. *Frontiers in Medicine* 2021; **7**.

67. Yue H, Bai X, Wang J, et al. Clinical characteristics of coronavirus disease 2019 in Gansu province, China. *Annals of Palliative Medicine* 2020; **9**(4): 1404-12.

68. Han Y, Luo Z, Zhai W, et al. Comparison of the clinical manifestations between different age groups of patients with overseas imported COVID-19. *PLOS ONE* 2020; **15**(12): e0243347.

69. Huang Q, Deng X, Li Y, et al. Clinical characteristics and drug therapies in patients with the common-type coronavirus disease 2019 in Hunan, China. *International Journal of Clinical Pharmacy* 2020; **42**(3): 837-45.

70. Zhai W, Luo Z, Zheng Y, et al. Moderate vs. mild cases of overseas-imported COVID-19 in Beijing: a retrospective cohort study. *Scientific Reports* 2021; **11**(1): 6483.

71. Jin X, Lian J-S, Hu J-H, et al. Epidemiological, clinical and virological characteristics of 74 cases of coronavirus-infected disease 2019 (COVID-19) with gastrointestinal symptoms. *Gut* 2020; **69**(6): 1002.

72. Uğurlu Ilgın B, Akbulut Koyuncu İ M, Kızıltunç E. Effect of triple antimicrobial therapy on electrocardiography parameters in patients with mild-to-moderate coronavirus disease 2019. *Anatol J Cardiol* 2021; **25**(3): 184-90.

73. Li Q, Li W, Jin Y, et al. Efficacy Evaluation of Early, Low-Dose, Short-Term Corticosteroids in Adults Hospitalized with Non-Severe COVID-19 Pneumonia: A Retrospective Cohort Study. *Infect Dis Ther* 2020; **9**(4): 823-36.

74. Wang L, Duan Y, Zhang W, et al. Epidemiologic and Clinical Characteristics of 26 Cases of COVID-19 Arising from Patient-to-Patient Transmission in Liaocheng, China. *Clin Epidemiol* 2020; **12**: 387-91.

75. Qi L, Yang Y, Jiang D, et al. Factors associated with the duration of viral shedding in adults with COVID-19 outside of Wuhan, China: a retrospective cohort study. *Int J Infect Dis* 2020; **96**: 531-7.

76. Yan X, Han X, Peng D, et al. Clinical Characteristics and Prognosis of 218 Patients With COVID-19: A Retrospective Study Based on Clinical Classification. *Front Med (Lausanne)* 2020; **7**: 485.

77. Dong X, Cao Y-y, Lu X-x, et al. Eleven faces of coronavirus disease 2019. *Allergy* 2020; **75**(7): 1699-709.

78. Liu H, Gao J, Wang Y, et al. Epidemiological and clinical characteristics of 2019 novel coronavirus disease (COVID-19) in Jilin, China: A descriptive study. *Medicine (Baltimore)* 2020; **99**(47): e23407.

79. Roozbeh F, Saeedi M, Alizadeh-Navaei R, et al. Sofosbuvir and daclatasvir for the treatment of COVID-19 outpatients: a double-blind, randomized controlled trial. *Journal of Antimicrobial Chemotherapy* 2021; **76**(3): 753-7.

80. Guo T, Shen Q, Zhou Z, et al. Combined Interventions for Severe Novel Coronavirus Disease (COVID-19): Experience from 350 Patients. *Infect Drug Resist* 2020; **13**: 3907-18.

81. Guan J, Wei X, Qin S, et al. Continuous tracking of COVID-19 patients' immune status. *Int Immunopharmacol* 2020; **89**(Pt A): 107034.

82. Huang R, Zhu L, Wang J, et al. Clinical Features of Patients With COVID-19 With Nonalcoholic Fatty Liver Disease. *Hepatology Communications* 2020; **4**(12): 1758-68.

83. Weidai Z, Senrong L, Jinfeng Z, et al. 低钠血症与新型冠状病毒肺炎患者重症化的关系研究. *中华危重病急救医学* 2020; **32**(7): 774-8.

84. Cheng F, Li Q, Han Y, et al. Analysis of Influencing Factors and Pharmaceutical Care of Patients with COVID-19 in Fangcang Shelter Hospital. *Infect Drug Resist* 2020; **13**: 3443-50.

85. Huang R, Zhu L, Xue L, et al. Clinical findings of patients with coronavirus disease 2019 in Jiangsu province, China: A retrospective, multi-center study. *PLoS Negl Trop Dis* 2020; **14**(5): e0008280.

86. Hu W, Chen X, He B, et al. Clinical characteristics of 16 patients with COVID-19 infection outside of Wuhan, China: a retrospective, single-center study. *Annals of Translational Medicine* 2020; **8**(10): 642.

87. Mancilla-Galindo J, García-Méndez J, Márquez-Sánchez J, et al. All-cause mortality among patients treated with repurposed antivirals and antibiotics for COVID-19 in Mexico City: A real-world observational study. *Excli j* 2021; **20**: 199-222.

88. Zuo Y, Liu Y, Zhong Q, Zhang K, Xu Y, Wang Z. Lopinavir/ritonavir and interferon combination therapy may help shorten the duration of viral shedding in patients with COVID-19: A retrospective study in two designated hospitals in Anhui, China. *J Med Virol* 2020; **92**(11): 2666-74.

89. Lin L, Jiang X, Zhang Z, et al. Gastrointestinal symptoms of 95 cases with SARS-CoV-2 infection. *Gut* 2020; **69**(6): 997.

90. Liu BM, Yang QQ, Zhao LY, Xie W, Si XY. Epidemiological characteristics of COVID-19 patients in convalescence period. *Epidemiol Infect* 2020; **148**: e108.

91. Chen Z, Hu J, Zhang Z, et al. Efficacy of hydroxychloroquine in patients with COVID-19: results of a randomized clinical trial. *medRxiv* 2020: 2020.03.22.20040758.

92. Demir E, Uyar M, Parmaksiz E, et al. COVID-19 in kidney transplant recipients: A multicenter experience in Istanbul. *Transpl Infect Dis* 2020; **22**(5): e13371.

93. Chu M, Bian L, Huang J, Chen Y, Wu D, Wang H. Clinical characteristics and outcomes of 48 patients hospitalized for COVID-19 infection in Wuxi: A retrospective cohort study. *Medicine* 2021; **100**(5).

94. Zhang J, Yu M, Tong S, Liu L-Y, Tang L-V. Predictive factors for disease progression in hospitalized patients with coronavirus disease 2019 in Wuhan, China. *Journal of Clinical Virology* 2020; **127**: 104392.

95. Xiaofang GC, Ma, Yujie M, Xiaoqi W, et al. 不同抗病毒方案对新型冠状病毒肺炎患者的临床疗效和安全性观察. *中华危重病急救医学* 2021; **32**(12): 1423-7.

96. Wang C, Zhou L, Chen J, et al. The differences of clinical characteristics and outcomes between imported and local patients of COVID-19 in Hunan: a two-center retrospective study. *Respiratory Research* 2020; **21**(1): 313.

97. Lu J, Yin Q, Li Q, et al. Clinical characteristics and factors affecting the duration of positive nucleic acid test for patients of COVID-19 in XinYu, China. *Journal of Clinical Laboratory Analysis* 2020; **34**(10): e23534.

98. Cheng LL, Guan WJ, Duan CY, et al. Effect of Recombinant Human Granulocyte Colony-Stimulating Factor for Patients With Coronavirus Disease 2019 (COVID-19) and Lymphopenia: A Randomized Clinical Trial. *JAMA Intern Med* 2021; **181**(1): 71-8.

99. Hong L, Ye E, Sun G, et al. Clinical and radiographic characteristics, management and short-term outcomes of patients with COVID-19 in Wenzhou, China. *BMC Infectious Diseases* 2020; **20**(1): 841.

100. Ting G, Yangling X, Xiaopeng H, et al. 40例新型冠状病毒肺炎的流行病学及临床特征分析. *中国呼吸与危重监护杂志* 2020; **9**(2): 148-53.

101. Huimin M, Yanyan W, Chao T, et al. 40例新型冠状病毒肺炎治愈患者的药物治疗分析. *中国药物应用与监测* 2020; **17**(3): 191-4.

102. Guo T, Shen Q, Ouyang X, et al. Clinical Findings in Diabetes Mellitus Patients with COVID-19. *J Diabetes Res* 2021; **2021**: 7830136.

103. Shalimar, Vaishnav M, Elhence A, et al. Outcome of Conservative Therapy in Coronavirus disease-2019 Patients Presenting With Gastrointestinal Bleeding. *Journal of Clinical and Experimental Hepatology* 2021; **11**(3): 327-33.

104. Luo H, Liu S, Wang Y, et al. Age differences in clinical features and outcomes in patients with COVID-19, Jiangsu, China: a retrospective, multicentre cohort study. *BMJ Open* 2020; **10**(10): e039887.

105. Jingru J, Zhengmao ZZ, Rao Donghua, Xu Pinxiang, Fu , Hong Q, Sufang W, Zongli Z, Zhongji Y. 上饶市45例新型冠状病毒肺炎患者的临床特征分析及其防治对策. *抗感染药学* 2020; **17**(12): 1787-90.

106. Tudoran C, Tudoran M, Lazureanu VE, et al. Evidence of Pulmonary Hypertension after SARS-CoV-2 Infection in Subjects without Previous Significant Cardiovascular Pathology. *J Clin Med* 2021; **10**(2).

107. Yitao Z, Mu C, Ling Z, et al. Predictors of clinical deterioration in non-severe patients with COVID-19: a retrospective cohort study. *Curr Med Res Opin* 2021; **37**(3): 385-91.

108. Wu J, Liu J, Zhao X, et al. Clinical Characteristics of Imported Cases of Coronavirus Disease 2019 (COVID-19) in Jiangsu Province: A Multicenter Descriptive Study. *Clin Infect Dis* 2020; **71**(15): 706-12.

109. Ma Y, Zeng H, Zhan Z, et al. Corticosteroid Use in the Treatment of COVID-19: A Multicenter Retrospective Study in Hunan, China. *Frontiers in Pharmacology* 2020; **11**.

110. Zhang H, Adu IK. Clinical characteristics of inflammatory indicators and therapeutic outcome of integrated chinese and western medicine in COVID-19 patients. *Acta Medica Mediterranea* 2020; **36**: 3901-6.

111. Deng L-S, Yuan J, Ding L, et al. Comparison of patients hospitalized with COVID-19, H7N9 and H1N1. *Infectious Diseases of Poverty* 2020; **9**(1): 163.

112. Wang S, Chen Z, Lin Y, et al. Clinical characteristics of 199 discharged patients with COVID-19 in Fujian Province: A multicenter retrospective study between January 22nd and February 27th, 2020. *PLOS ONE* 2020; **15**(11): e0242307.

113. Zhang D, Zhu Z, Bi J, et al. Clinical Characteristics and Risk Factors in Coronavirus Disease 2019 Patients with Liver Injury. *Med Sci Monit* 2020; **26**: e928849.

114. Gupta ML, Gothwal S, Gupta RK, et al. Duration of Viral Clearance in Children With SARS-CoV-2 Infection in Rajasthan, India. *Indian Pediatr* 2021; **58**(2): 123-5.

115. Wan S, Xiang Y, Fang W, et al. Clinical features and treatment of COVID-19 patients in northeast Chongqing. *J Med Virol* 2020; **92**(7): 797-806.

116. Plavunov N, Kadyshev V, Sidorov A, Proskurina L, Goncharova N. Cutaneous Manifestations in Patients with Covid-19 in the Practice of Emergency Medical Care. *The Russian Archives of Internal Medicine* 2020; **10**: 223-9.

117. Lai C, Yu R, Wang M, et al. Shorter incubation period is associated with severe disease progression in patients with COVID-19. *Virulence* 2020; **11**(1): 1443-52.

118. Guan W-j, Ni Z-y, Hu Y, et al. Clinical Characteristics of Coronavirus Disease 2019 in China. *New England Journal of Medicine* 2020; **382**(18): 1708-20.

119. Abdulrahman A, AlSayed I, AlMadhi M, et al. The Efficacy and Safety of Hydroxychloroquine in Patients with COVID-19: A Multicenter National Retrospective Cohort. *Infect Dis Ther* 2021; **10**(1): 439-55.

120. Cai Q, Huang D, Ou P, et al. COVID-19 in a designated infectious diseases hospital outside Hubei Province, China. *Allergy* 2020; **75**(7): 1742-52.

121. Zhang Q, Wang Z, Lv Y, et al. Clinical features and prognostic factors of patients with COVID-19 in Henan Province, China. *Human Cell* 2021; **34**(2): 419-35.

122. Shao L, Li X, Zhou Y, et al. Novel Insights Into Illness Progression and Risk Profiles for Mortality in Non-survivors of COVID-19. *Front Med (Lausanne)* 2020; **7**: 246.

123. Saha A, Ahsan MM, Quader MT-U, et al. Clinical characteristics and outcomes of COVID-19 infected diabetic patients admitted in ICUs of the southern region of Bangladesh. *Diabetes & Metabolic Syndrome: Clinical Research & Reviews* 2021; **15**(1): 229-35.

124. Gao Q, Hu Y, Dai Z, Xiao F, Wang J, Wu J. The epidemiological characteristics of 2019 novel coronavirus diseases (COVID-19) in Jingmen, Hubei, China. *Medicine (Baltimore)* 2020; **99**(23): e20605.

125. Jin L, Tang W, Song L, et al. Acute cardiac injury in adult hospitalized COVID-19 patients in Zhuhai, China. *Cardiovascular Diagnosis and Therapy* 2020; **10**(5): 1303-12.

126. Qingrong W, Xiaobing S, Xiangwen G, Chunyun L, Jianping L, Hui L. 新型冠状病毒肺炎患者55例临床分析. *江苏医药* 2020; **46**(6): 546-50.

127. Ye H, Zhao C, Yang L, et al. Twelve out of 117 recovered COVID-19 patients retest positive in a single-center study of China. *eClinicalMedicine* 2020; **26**.

128. Li Y, Li J, Ke J, et al. Adverse Outcomes Associated With Corticosteroid Use in Critical COVID-19: A Retrospective Multicenter Cohort Study. *Frontiers in Medicine* 2021; **8**.

129. Zhang H, Shang W, Liu Q, Zhang X, Zheng M, Yue M. Clinical characteristics of 194 cases of COVID-19 in Huanggang and Taian, China. *Infection* 2020; **48**(5): 687-94.

130. Yangling X, Lizhen W, Wenming Z, Ting G, Changgang W. 新型冠状病毒肺炎35例流行病学及临床特征分析. *临床肺科杂志* 2020; **25**(7): 1082-6.

131. Lei M, Lin K, Pi Y, et al. Clinical Features and Risk Factors of ICU Admission for COVID-19 Patients with Diabetes. *J Diabetes Res* 2020; **2020**: 5237840.

132. Tian J, Yan S, Wang H, et al. Hanshiyi Formula, a medicine for Sars-CoV2 infection in China, reduced the proportion of mild and moderate COVID-19 patients turning to severe status: A cohort study. *Pharmacol Res* 2020; **161**: 105127.

133. Fatima SA, Asif M, Khan KA, Siddique N, Khan AZ. Comparison of efficacy of dexamethasone and methylprednisolone in moderate to severe covid 19 disease. *Ann Med Surg (Lond)* 2020; **60**: 413-6.

134. Changquan L, Yuejun P, Xilong D, et al. 广州278例新型冠状病毒肺炎患者的流行病学、临床特征及治疗分析. *中华内科杂志* 2020; **59**(8): 598-604.

135. Liu Y, Ding N, Zhou S, et al. Comparison of clinical characteristics between patients with coronavirus disease 2019 (COVID-19) who retested RT-PCR positive versus negative: a retrospective study of data from Nanjing. *J Thorac Dis* 2020; **12**(11): 6435-45.

136. Chen X, Zhu B, Hong W, et al. Associations of clinical characteristics and treatment regimens with the duration of viral RNA shedding in patients with COVID-19. *International Journal of Infectious Diseases* 2020; **98**: 252-60.

137. Huang C, Wang Y, Li X, et al. Clinical features of patients infected with 2019 novel coronavirus in Wuhan, China. *The Lancet* 2020; **395**(10223): 497-506.

138. Fan Z, Chen L, Li J, et al. Clinical Features of COVID-19-Related Liver Functional Abnormality. *Clinical Gastroenterology and Hepatology* 2020; **18**(7): 1561-6.

139. Zheng T, Yang C, Wang H-Y, et al. Clinical characteristics and outcomes of COVID-19 patients with gastrointestinal symptoms admitted to Jianghan Fangcang Shelter Hospital in Wuhan, China. *Journal of Medical Virology* 2020; **92**(11): 2735-41.

140. Cavalcanti AB, Zampieri FG, Rosa RG, et al. Hydroxychloroquine with or without Azithromycin in Mild-to-Moderate Covid-19. *New England Journal of Medicine* 2020; **383**(21): 2041-52.

141. Tanriverdİ E, Çörtük M, Çörtük M, et al. Hydroxychloroquine plus azithromycin and early hospital admission are beneficial in COVID-19 patients: Turkish experience with real-life data. *Turk J Med Sci* 2021; **51**(1): 10-5.

142. Wang F, Hou H, Wang T, et al. Establishing a model for predicting the outcome of COVID-19 based on combination of laboratory tests. *Travel Medicine and Infectious Disease* 2020; **36**: 101782.

143. Qing C, Tao H, Ming Y, et al. 成都市不同年龄段新型冠状病毒肺炎患者临床特征及预后分析. *实用心脑肺血管病杂志* 2020; **28**(12): 9-15.

144. Xie Q, Fan F, Fan X-P, et al. COVID-19 patients managed in psychiatric inpatient settings due to first-episode mental disorders in Wuhan, China: clinical characteristics, treatments, outcomes, and our experiences. *Translational Psychiatry* 2020; **10**(1): 337.

145. Li R, Liu G, Huang X, et al. Dynamic changes in clinical and CT characteristics of COVID-19 cases with different exposure histories: a retrospective study. *BMC Infectious Diseases* 2020; **20**(1): 567.

146. Molla MMA, Yeasmin M, Islam MK, et al. Antibiotic Prescribing Patterns at COVID-19 Dedicated Wards in Bangladesh: Findings from a Single Center Study. *Infection Prevention in Practice* 2021; **3**(2): 100134.

147. Sekhavati E, Jafari F, SeyedAlinaghi S, et al. Safety and effectiveness of azithromycin in patients with COVID-19: An open-label randomised trial. *International Journal of Antimicrobial Agents* 2020; **56**(4): 106143.

148. Ersan G, Bac G, Yuksel O, et al. The Demographic and Clinical Features of 479 COVID-19 Patients: A Single-Center Experience. *Mediterranean Journal of Infection Microbes and Antimicrobials* 2020.

149. Ji M, Yuan L, Shen W, et al. Characteristics of disease progress in patients with coronavirus disease 2019 in Wuhan, China. *Epidemiology and Infection* 2020; **148**: e94.

150. Wu C, Chen X, Cai Y, et al. Risk Factors Associated With Acute Respiratory Distress Syndrome and Death in Patients With Coronavirus Disease 2019 Pneumonia in Wuhan, China. *JAMA Intern Med* 2020; **180**(7): 934-43.

151. Borba MGS, Val FFA, Sampaio VS, et al. Effect of High vs Low Doses of Chloroquine Diphosphate as Adjunctive Therapy for Patients Hospitalized With Severe Acute Respiratory Syndrome Coronavirus 2 (SARS-CoV-2) Infection: A Randomized Clinical Trial. *JAMA Network Open* 2020; **3**(4): e208857-e.

152. Zhang W, Li L, Liu J, et al. The characteristics and predictive role of lymphocyte subsets in COVID-19 patients. *International Journal of Infectious Diseases* 2020; **99**: 92-9.

153. Yan J, Guo J, Fan C, et al. Coronavirus disease 2019 in pregnant women: a report based on 116 cases. *Am J Obstet Gynecol* 2020; **223**(1): 111.e1-.e14.

154. Martinez-Guerra BA, Gonzalez-Lara MF, de-Leon-Cividanes NA, et al. Antimicrobial Resistance Patterns and Antibiotic Use during Hospital Conversion in the COVID-19 Pandemic. *Antibiotics (Basel)* 2021; **10**(2).

155. Yao Q, Wang P, Wang X, et al. A retrospective study of risk factors for severe acute respiratory syndrome coronavirus 2 infections in hospitalized adult patients. *Pol Arch Intern Med* 2020; **130**(5): 390-9.

156. Junhua M, Yang H, Qian C, Qiang G, Yonggang C, Jing A. 清肺排毒汤治疗普通型/重型新型冠状病毒肺炎的回顾性研究. *中国医院药学杂志* 2020; **40**(20): 2152-7.

157. Jiangjun W, Yu Z, Zhicang Z. 分析40例新型冠状病毒肺炎危重症病例临床特点及药物治疗方案. *世界最新医学信息文摘* 2020; **20**(68): 160-2.

158. Fan C, Tiying D, Yan G, et al. 新型冠状病毒肺炎患者抗菌药物使用情况分析. *医药导报* 2020; **39**(9): 1285-8.

159. Feng Y, Ling Y, Bai T, et al. COVID-19 with Different Severities: A Multicenter Study of Clinical Features. *Am J Respir Crit Care Med* 2020; **201**(11): 1380-8.

160. Wei X, Hairong Z, Xioyan W, et al. 新型冠状病毒肺炎患者入院时临床特征及致死亡危险因素分析. *武汉大学学报(医学版)* 2021; **42**(1): 6-9.

161. Zhanxiang L, Yingming F, Jingyi O, et al. 新型冠状病毒肺炎合并细菌及真菌感染的诊疗特点. *实用医学杂志* 2021; **37**(1): 16-9.

162. Shenghao W, Jianping W, Shijun W, et al. 探讨新型冠状病毒合并肺炎支原体感染患者的临床特征. *心肺血管病杂志* 2020; **40**(1): 29-36.

163. Galan LEB, Santos NMD, Asato MS, et al. Phase 2 randomized study on chloroquine, hydroxychloroquine or ivermectin in hospitalized patients with severe manifestations of SARS-CoV-2 infection. *Pathog Glob Health* 2021; **115**(4): 235-42.

164. Liu D, Yang Q, Chen W, et al. Troponin I, a risk factor indicating more severe pneumonia among patients with novel coronavirus infected pneumonia. *Clinical Infection in Practice* 2020; **7-8**: 100037.

165. Pan L, Mu M, Yang P, et al. Clinical Characteristics of COVID-19 Patients With Digestive Symptoms in Hubei, China: A Descriptive, Cross-Sectional, Multicenter Study. *Am J Gastroenterol* 2020; **115**(5): 766-73.

166. Peng H, Gao P, Xu Q, et al. Coronavirus disease 2019 in children: Characteristics, antimicrobial treatment, and outcomes. *Journal of Clinical Virology* 2020; **128**: 104425.

167. Yi P, Yang X, Ding C, et al. Risk factors and clinical features of deterioration in COVID-19 patients in Zhejiang, China: a single-centre, retrospective study. *BMC Infectious Diseases* 2020; **20**(1): 943.

168. Li C, Luo F, Liu C, et al. Effect of a genetically engineered interferon-alpha versus traditional interferon-alpha in the treatment of moderate-to-severe COVID-19: a randomised clinical trial. *Annals of Medicine* 2021; **53**(1): 391-401.

169. Xie S, Zhang G, Yu H, et al. The epidemiologic and clinical features of suspected and confirmed cases of imported 2019 novel coronavirus pneumonia in north Shanghai, China. *Ann Transl Med* 2020; **8**(10): 637.

170. Shi N, Guo L, Liu B, et al. Efficacy and safety of Chinese herbal medicine versus Lopinavir-Ritonavir in adult patients with coronavirus disease 2019: A non-randomized controlled trial. *Phytomedicine* 2021; **81**: 153367.

171. Peymani P, Dehesh T, Aligolighasemabadi F, et al. Statins in patients with COVID-19: a retrospective cohort study in Iranian COVID-19 patients. *Transl Med Commun* 2021; **6**(1): 3.

172. Ling L, Yulai Y, Suhua A, et al. 武汉地区94例普通型与重型、危重型COVID-19患者临床特征比较. *重庆医科大学学报* 2020; **45**(7): 929-36.

173. Tong S, Su Y, Yu Y, et al. Ribavirin therapy for severe COVID-19: a retrospective cohort study. *Int J Antimicrob Agents* 2020; **56**(3): 106114.

174. Akbariqomi M, Hosseini MS, Rashidiani J, et al. Clinical characteristics and outcome of hospitalized COVID-19 patients with diabetes: A single-center, retrospective study in Iran. *Diabetes Research and Clinical Practice* 2020; **169**: 108467.

175. Wu H, Zhu H, Yuan C, et al. Clinical and Immune Features of Hospitalized Pediatric Patients With Coronavirus Disease 2019 (COVID-19) in Wuhan, China. *JAMA Network Open* 2020; **3**(6): e2010895-e.

176. Malekzadeh R, Abedini A, Mohsenpour B, et al. Subcutaneous tocilizumab in adults with severe and critical COVID-19: A prospective open-label uncontrolled multicenter trial. *Int Immunopharmacol* 2020; **89**(Pt B): 107102.

177. Wang Z, Yang B, Li Q, Wen L, Zhang R. Clinical Features of 69 Cases With Coronavirus Disease 2019 in Wuhan, China. *Clin Infect Dis* 2020; **71**(15): 769-77.

178. Cheng B, Hu J, Zuo X, et al. Predictors of progression from moderate to severe coronavirus disease 2019: a retrospective cohort. *Clinical Microbiology and Infection* 2020; **26**(10): 1400-5.

179. de Melo AC, Thuler LCS, da Silva JL, et al. Cancer inpatients with COVID-19: A report from the Brazilian National Cancer Institute. *PLOS ONE* 2020; **15**(10): e0241261.

180. Chen N, Zhou M, Dong X, et al. Epidemiological and clinical characteristics of 99 cases of 2019 novel coronavirus pneumonia in Wuhan, China: a descriptive study. *The Lancet* 2020; **395**(10223): 507-13.

181. Najafi A, Ghanei M, Janbabaei G, et al. Real Clinical Practice and Therapeutic Management Following COVID-19 Crisis in two Hospitals in Iran: A Statistical and Conceptual View. *Tanaffos* 2020; **19**(2): 112-21.

182. Zhang B, Xie R, Hubert SM, et al. Characteristics and Outcomes of 35 Breast Cancer Patients Infected With COVID-19. *Front Oncol* 2020; **10**: 570130.

183. Wang D, Hu B, Hu C, et al. Clinical Characteristics of 138 Hospitalized Patients With 2019 Novel Coronavirus–Infected Pneumonia in Wuhan, China. *JAMA* 2020; **323**(11): 1061-9.

184. Xiao M, Tian J, Zhou Y, et al. Efficacy of Huoxiang Zhengqi dropping pills and Lianhua Qingwen granules in treatment of COVID-19: A randomized controlled trial. *Pharmacol Res* 2020; **161**: 105126.

185. Xu G, Liu F, Ye M, et al. No Evidence of Re-infection or Person-to-Person Transmission in Cured COVID-19 Patients in Guangzhou, a Retrospective Observational Study. *Frontiers in Medicine* 2020; **7**.

186. Yang L, Liu J, Zhang R, et al. Epidemiological and clinical features of 200 hospitalized patients with corona virus disease 2019 outside Wuhan, China: A descriptive study. *J Clin Virol* 2020; **129**: 104475.

187. Zhao J, Gao H-Y, Feng Z-Y, Wu Q-J. A Retrospective Analysis of the Clinical and Epidemiological Characteristics of COVID-19 Patients in Henan Provincial People's Hospital, Zhengzhou, China. *Frontiers in Medicine* 2020; **7**.

188. Pei G, Zhang Z, Peng J, et al. Renal Involvement and Early Prognosis in Patients with COVID-19 Pneumonia. *Journal of the American Society of Nephrology* 2020; **31**(6).

189. Chen X, Chen Y, Wu C, et al. Coagulopathy is a major extrapulmonary risk factor for mortality in hospitalized patients with COVID-19 with type 2 diabetes. *BMJ Open Diabetes Research &amp;amp; Care* 2020; **8**(2): e001851.

190. Hu J, Zhang Y, Wang W, et al. Clinical characteristics of 14 COVID-19 deaths in Tianmen, China: a single-center retrospective study. *BMC Infect Dis* 2021; **21**(1): 88.

191. Xu Y, Xu Z, Liu X, et al. Clinical Findings of COVID-19 Patients Admitted to Intensive Care Units in Guangdong Province, China: A Multicenter, Retrospective, Observational Study. *Front Med (Lausanne)* 2020; **7**: 576457.

192. Bosevski M, Janusevski F, Kapsarov K. Utility of Combined Echocardiography and Lung Ultrasound for Coronavirus Disease-19 Intensive Care Unit Patients: Case Series. *Open Access Macedonian Journal of Medical Sciences* 2020.

193. Shu Z, Zhou Y, Chang K, et al. Clinical features and the traditional Chinese medicine therapeutic characteristics of 293 COVID-19 inpatient cases. *Front Med* 2020; **14**(6): 760-75.

194. Ñamendys-Silva SA, Alvarado-Ávila PE, Domínguez-Cherit G, et al. Outcomes of patients with COVID-19 in the intensive care unit in Mexico: A multicenter observational study. *Heart Lung* 2021; **50**(1): 28-32.

195. Sun M, Ruan X, Li Y, et al. Clinical characteristics of 30 COVID-19 patients with epilepsy: A retrospective study in Wuhan. *International Journal of Infectious Diseases* 2021; **103**: 647-53.

196. Solaymani-Dodaran M, Ghanei M, Bagheri M, et al. Safety and efficacy of Favipiravir in moderate to severe SARS-CoV-2 pneumonia. *Int Immunopharmacol* 2021; **95**: 107522.

197. Chen W, Li Z, Yang B, et al. Delayed-phase thrombocytopenia in patients with coronavirus disease 2019 (COVID-19). *British Journal of Haematology* 2020; **190**(2): 179-84.

198. Sadeghi A, Ali Asgari A, Norouzi A, et al. Sofosbuvir and daclatasvir compared with standard of care in the treatment of patients admitted to hospital with moderate or severe coronavirus infection (COVID-19): a randomized controlled trial. *Journal of Antimicrobial Chemotherapy* 2020; **75**(11): 3379-85.

199. Cao B, Wang Y, Wen D, et al. A Trial of Lopinavir–Ritonavir in Adults Hospitalized with Severe Covid-19. *New England Journal of Medicine* 2020; **382**(19): 1787-99.

200. Zhang S, Xu Y, Li J, et al. Symptomless multi-variable apnea prediction index assesses adverse outcomes in patients with Corona Virus Disease 2019. *Sleep Med* 2020; **75**: 294-300.

201. Wang Y, Lu C, Li H, et al. Efficacy and safety assessment of severe COVID-19 patients with Chinese medicine: A retrospective case series study at early stage of the COVID-19 epidemic in Wuhan, China. *Journal of Ethnopharmacology* 2021; **277**: 113888.

202. Peng L, Lv QQ, Yang F, et al. The interval between onset and admission predicts disease progression in COVID-19 patients. *Ann Transl Med* 2021; **9**(3): 213.

203. Davoudi-Monfared E, Rahmani H, Khalili H, et al. A Randomized Clinical Trial of the Efficacy and Safety of Interferon β-1a in Treatment of Severe COVID-19. *Antimicrobial Agents and Chemotherapy* 2020; **64**(9): e01061-20.

204. Fang C, Qiang L, Fang Z, et al. 方舱医院290名新型冠状病毒肺炎患者用药现状分析与建议. *中国医院药学杂志* 2020; **40**(11): 1189-91.

205. Li HY, Wang JW, Xu LW, Zhao XL, Feng JX, Xu YZ. Clinical analysis of 132 cases COVID-19 from Wuhan. *Medicine (Baltimore)* 2020; **99**(44): e22847.

206. Chen Y, Zhang K, Zhu G, et al. Clinical characteristics and treatment of critically ill patients with COVID-19 in Hebei. *Annals of Palliative Medicine* 2020; **9**(4): 2118-30.

207. Li Q, Chen L, Li Q, et al. Cancer increases risk of in-hospital death from COVID-19 in persons <65 years and those not in complete remission. *Leukemia* 2020; **34**(9): 2384-91.

208. Tang Y, Li Y, Sun J, Pan H, Yao F, Jiao X. Selection of an Optimal Combination Panel to Better Triage COVID-19 Hospitalized Patients. *J Inflamm Res* 2020; **13**: 773-87.

209. Marcolino MS, Ziegelmann PK, Souza-Silva MVR, et al. Clinical characteristics and outcomes of patients hospitalized with COVID-19 in Brazil: Results from the Brazilian COVID-19 registry. *International Journal of Infectious Diseases* 2021; **107**: 300-10.

210. Li J, Liu Z, Wu G, et al. D-Dimer as a Prognostic Indicator in Critically Ill Patients Hospitalized With COVID-19 in Leishenshan Hospital, Wuhan, China. *Front Pharmacol* 2020; **11**: 600592.

211. Zhou S, Mi S, Luo S, et al. Risk Factors for Mortality in 220 Patients With COVID-19 in Wuhan, China: A Single-Center, Retrospective Study. *Ear, Nose & Throat Journal* 2020; **100**(2_suppl): 140S-7S.

212. Molaei H, Khedmat L, Nemati E, Rostami Z, Saadat SH. Iranian kidney transplant recipients with COVID-19 infection: Clinical outcomes and cytomegalovirus coinfection. *Transplant Infectious Disease* 2021; **23**(1): e13455.

213. Du H, Dong X, Zhang J-j, et al. Clinical characteristics of 182 pediatric COVID-19 patients with different severities and allergic status. *Allergy* 2021; **76**(2): 510-32.

214. Yang Q, Xie L, Zhang W, et al. Analysis of the clinical characteristics, drug treatments and prognoses of 136 patients with coronavirus disease 2019. *Journal of Clinical Pharmacy and Therapeutics* 2020; **45**(4): 609-16.

215. Lima-Setta F, Magalhães-Barbosa MCd, Rodrigues-Santos G, et al. Multisystem inflammatory syndrome in children (MIS-C) during SARS-CoV-2 pandemic in Brazil: a multicenter, prospective cohort study. *Jornal de Pediatria* 2021; **97**(3): 354-61.

216. Ramadan HK, Mahmoud MA, Aburahma MZ, et al. Predictors of Severity and Co-Infection Resistance Profile in COVID-19 Patients: First Report from Upper Egypt. *Infect Drug Resist* 2020; **13**: 3409-22.

217. Lian N, Xie H, Lin S, Huang J, Zhao J, Lin Q. Umifenovir treatment is not associated with improved outcomes in patients with coronavirus disease 2019: a retrospective study. *Clinical Microbiology and Infection* 2020; **26**(7): 917-21.

218. Zhao X, Li Y, Ge Y, et al. Evaluation of Nutrition Risk and Its Association With Mortality Risk in Severely and Critically Ill COVID-19 Patients. *Journal of Parenteral and Enteral Nutrition* 2021; **45**(1): 32-42.

219. Ding X, Zhang J, Liu L, et al. High-density lipoprotein cholesterol as a factor affecting virus clearance in covid-19 patients. *Respir Med* 2020; **175**: 106218.

220. Wu C, Hou D, Du C, et al. Corticosteroid therapy for coronavirus disease 2019-related acute respiratory distress syndrome: a cohort study with propensity score analysis. *Critical Care* 2020; **24**(1): 643.

221. Cen Y, Chen X, Shen Y, et al. Risk factors for disease progression in patients with mild to moderate coronavirus disease 2019-a multi-centre observational study. *Clin Microbiol Infect* 2020; **26**(9): 1242-7.

222. Wang J, Zheng X, Chen J. Clinical progression and outcomes of 260 patients with severe COVID-19: an observational study. *Sci Rep* 2021; **11**(1): 3166.

223. Liu C, Wen Y, Wan W, Lei J, Jiang X. Clinical characteristics and antibiotics treatment in suspected bacterial infection patients with COVID-19. *International Immunopharmacology* 2021; **90**: 107157.

224. Chengyi HU, Lushan X, Hongbo Z, et al. Effect of hypertension on outcomes of patients with COVID-19. *Nan Fang Yi Ke Da Xue Xue Bao* 2020; **40**(11): 1537-42.

225. Ussaid A, Riaz B, Rafai W, et al. Clinical Characteristics of 47 Death Cases With COVID-19: A Retrospective Study at a Tertiary Center in Lahore. *Cureus* 2020; **12**(12): e12039.

226. Davidescu I, Odajiu I, Ilie M, et al. Influence of tocilizumab on the outcome of patients with covid-19. Retrospective observational study. *Farmacia* 2020; **68**: 792-9.

227. Wu J, Li J, Zhu G, et al. Clinical Features of Maintenance Hemodialysis Patients with 2019 Novel Coronavirus-Infected Pneumonia in Wuhan, China. *Clin J Am Soc Nephrol* 2020; **15**(8): 1139-45.

228. Chen J, Bai H, Liu J, et al. Distinct Clinical Characteristics and Risk Factors for Mortality in Female Inpatients With Coronavirus Disease 2019 (COVID-19): A Sex-stratified, Large-scale Cohort Study in Wuhan, China. *Clinical Infectious Diseases* 2020; **71**(12): 3188-95.

229. Wang J, Wang Z, Zhu Y, et al. Identify the Risk Factors of COVID-19-Related Acute Kidney Injury: A Single-Center, Retrospective Cohort Study. *Front Med (Lausanne)* 2020; **7**: 436.

230. Sujuan Z, Xiankun W, Yanli X, et al. 境外输入性新型冠状病毒肺炎69例的流行病学及临床特征分析. *中华传染病杂志* 2020; **38**(11): 690-5.

231. Cheng Y, Luo R, Wang X, et al. The Incidence, Risk Factors, and Prognosis of Acute Kidney Injury in Adult Patients with Coronavirus Disease 2019. *Clinical Journal of the American Society of Nephrology* 2020; **15**(10).

232. Shi S, Qin M, Cai Y, et al. Characteristics and clinical significance of myocardial injury in patients with severe coronavirus disease 2019. *European Heart Journal* 2020; **41**(22): 2070-9.

233. Yang K, Sheng Y, Huang C, et al. Clinical characteristics, outcomes, and risk factors for mortality in patients with cancer and COVID-19 in Hubei, China: a multicentre, retrospective, cohort study. *The Lancet Oncology* 2020; **21**(7): 904-13.

234. Furtado RHM, Berwanger O, Fonseca HA, et al. Azithromycin in addition to standard of care versus standard of care alone in the treatment of patients admitted to the hospital with severe COVID-19 in Brazil (COALITION II): a randomised clinical trial. *The Lancet* 2020; **396**(10256): 959-67.

235. Cao Y, Wei J, Zou L, et al. Ruxolitinib in treatment of severe coronavirus disease 2019 (COVID-19): A multicenter, single-blind, randomized controlled trial. *J Allergy Clin Immunol* 2020; **146**(1): 137-46.e3.

236. Peng X, Chen Y, Deng L, et al. Clinical features of critically ill patients infected with SARS-CoV-2 outside Wuhan with and without diabetes. *Int J Diabetes Dev Ctries* 2020; **40**(4): 482-90.

237. Song J, Zeng M, Wang H, et al. Distinct effects of asthma and COPD comorbidity on disease expression and outcome in patients with COVID-19. *Allergy* 2021; **76**(2): 483-96.

238. Ma G-G, Shen Y-X, Wu L, et al. Effect of liver injury on prognosis and treatment of hospitalized patients with COVID-19 pneumonia. *Annals of Translational Medicine* 2020; **9**(1): 10.

239. Salehi M, Ahmadikia K, Mahmoudi S, et al. Oropharyngeal candidiasis in hospitalised COVID-19 patients from Iran: Species identification and antifungal susceptibility pattern. *Mycoses* 2020; **63**(8): 771-8.

240. Agarwal A, Mukherjee A, Kumar G, Chatterjee P, Bhatnagar T, Malhotra P. Convalescent plasma in the management of moderate covid-19 in adults in India: open label phase II multicentre randomised controlled trial (PLACID Trial). *Bmj* 2020; **371**: m3939.

241. Lian J, Jin X, Hao S, et al. Epidemiological, clinical, and virological characteristics of 465 hospitalized cases of coronavirus disease 2019 (COVID-19) from Zhejiang province in China. *Influenza Other Respir Viruses* 2020; **14**(5): 564-74.

242. Tian R, Wu W, Wang C, et al. Clinical characteristics and survival analysis in critical and non-critical patients with COVID-19 in Wuhan, China: a single-center retrospective case control study. *Scientific Reports* 2020; **10**(1): 17524.

243. Yu Y, Xu D, Fu S, et al. Patients with COVID-19 in 19 ICUs in Wuhan, China: a cross-sectional study. *Critical Care* 2020; **24**(1): 219.

244. Feng X, Li P, Ma L, et al. Clinical Characteristics and Short-Term Outcomes of Severe Patients With COVID-19 in Wuhan, China. *Frontiers in Medicine* 2020; **7**.

245. Huang Y, Chen Z, Wang Y, et al. Clinical characteristics of 17 patients with COVID-19 and systemic autoimmune diseases: a retrospective study. *Annals of the Rheumatic Diseases* 2020; **79**(9): 1163.

246. Ping Y, Wei A, Min C, et al. 110例新型冠状病毒肺炎出院患者抗菌药物使用情况分析. *中国药物警戒* 2020; **17**(6): 338-42.

247. Zhang Y, Cui Y, Shen M, et al. Association of diabetes mellitus with disease severity and prognosis in COVID-19: A retrospective cohort study. *Diabetes Research and Clinical Practice* 2020; **165**.

248. Limian L, Yunmi Y, Guangbin C, Yanlin J, Wenjing Q. 基于帕累托图的某院新型冠状病毒肺炎不合理用药医嘱分析. *中国医院用药评价与分析* 2020; **20**(11): 1383-6.

249. Chen F-f, Zhong M, Liu Y, et al. The characteristics and outcomes of 681 severe cases with COVID-19 in China. *Journal of Critical Care* 2020; **60**: 32-7.

250. Hui Y, Li Y, Tong X, et al. The risk factors for mortality of diabetic patients with severe COVID-19: A retrospective study of 167 severe COVID-19 cases in Wuhan. *PLoS One* 2020; **15**(12): e0243602.

251. Du Y, Tu L, Zhu P, et al. Clinical Features of 85 Fatal Cases of COVID-19 from Wuhan. A Retrospective Observational Study. *Am J Respir Crit Care Med* 2020; **201**(11): 1372-9.

252. Wang S, Zhang Q, Wang P, et al. Clinical features of hypertensive patients with COVID-19 compared with a normotensive group: Single-center experience in China. *Open Med (Wars)* 2021; **16**(1): 367-74.

253. Li J, Xu G, Yu H, Peng X, Luo Y, Cao Ca. Clinical Characteristics and Outcomes of 74 Patients With Severe or Critical COVID-19. *The American Journal of the Medical Sciences* 2020; **360**(3): 229-35.

254. Yu B, Li C, Sun Y, Wang DW. Insulin Treatment Is Associated with Increased Mortality in Patients with COVID-19 and Type 2 Diabetes. *Cell Metabolism* 2021; **33**(1): 65-77.e2.

255. Zheng Y, Sun LJ, Xu M, et al. Clinical characteristics of 34 COVID-19 patients admitted to intensive care unit in Hangzhou, China. *J Zhejiang Univ Sci B* 2020; **21**(5): 378-87.

256. Wang Z, Wang Z. Identification of risk factors for in-hospital death of COVID - 19 pneumonia -- lessions from the early outbreak. *BMC Infectious Diseases* 2021; **21**(1): 113.

257. Zhou Y, Han T, Chen J, et al. Clinical and Autoimmune Characteristics of Severe and Critical Cases of COVID-19. *Clinical and Translational Science* 2020; **13**(6): 1077-86.

258. Li J, Wang J, Yang Y, et al. Etiology and antimicrobial resistance of secondary bacterial infections in patients hospitalized with COVID-19 in Wuhan, China: a retrospective analysis. *Antimicrobial Resistance & Infection Control* 2020; **9**(1): 153.

259. Wang R, Kong L, Xu Q, et al. On-ward participation of clinical pharmacists in a Chinese intensive care unit for patients with COVID-19: A retrospective, observational study. *Res Social Adm Pharm* 2021; **17**(1): 1853-8.

260. Ji X-y, Ma Y, Shi N-n, et al. Clinical characteristics and treatment outcome of COVID-19 patients with stroke in China: A multicenter retrospective study. *Phytomedicine* 2021; **81**: 153433.

261. Liu Z, Li J, Huang J, et al. Association Between Diabetes and COVID-19: A Retrospective Observational Study With a Large Sample of 1,880 Cases in Leishenshan Hospital, Wuhan. *Frontiers in Endocrinology* 2020; **11**.

262. Xia P, Wen Y, Duan Y, et al. Clinicopathological Features and Outcomes of Acute Kidney Injury in Critically Ill COVID-19 with Prolonged Disease Course: A Retrospective Cohort. *Journal of the American Society of Nephrology* 2020; **31**(9).

263. Wang Z, Zhang D, Wang S, et al. A Retrospective Study from 2 Centers in China on the Effects of Continued Use of Angiotensin-Converting Enzyme Inhibitors and Angiotensin II Receptor Blockers in Patients with Hypertension and COVID-19. *Med Sci Monit* 2020; **26**: e926651.

264. Sharifipour E, Shams S, Esmkhani M, et al. Evaluation of bacterial co-infections of the respiratory tract in COVID-19 patients admitted to ICU. *BMC Infectious Diseases* 2020; **20**(1): 646.

265. Mannan A, Mehedi HMH, Chy N, et al. A multi-centre, cross-sectional study on coronavirus disease 2019 in Bangladesh: clinical epidemiology and short-term outcomes in recovered individuals. *New Microbes New Infect* 2021; **40**: 100838.

266. Wu F, Zhou Y, Wang Z, et al. Clinical characteristics of COVID-19 infection in chronic obstructive pulmonary disease: a multicenter, retrospective, observational study. *J Thorac Dis* 2020; **12**(5): 1811-23.

267. Wang ZH, Shu C, Ran X, Xie CH, Zhang L. Critically Ill Patients with Coronavirus Disease 2019 in a Designated ICU: Clinical Features and Predictors for Mortality. *Risk Manag Healthc Policy* 2020; **13**: 833-45.

268. Li P, Chen L, Liu Z, et al. Clinical features and short-term outcomes of elderly patients with COVID-19. *Int J Infect Dis* 2020; **97**: 245-50.

269. Temel S, Gundogan K, Ulger B, et al. Characteristics and Outcomes of the Patients Infected with SARS-CoV-2 Admitted to Intensive Care Units: Erciyes University COVID-19 Center Experience. *Erciyes Medical Journal* 2020; **42**: 436+.

270. Li Y, Wu J, Wang S, et al. Progression to fibrosing diffuse alveolar damage in a series of 30 minimally invasive autopsies with COVID-19 pneumonia in Wuhan, China. *Histopathology* 2021; **78**(4): 542-55.

271. Yu B, Li C, Chen P, et al. Low dose of hydroxychloroquine reduces fatality of critically ill patients with COVID-19. *Science China Life Sciences* 2020; **63**(10): 1515-21.

272. Dai Y, Liu Z, Du X, et al. Acute Kidney Injury in Hospitalized Patients Infected with COVID-19 from Wuhan, China: A Retrospective Study. *BioMed Research International* 2021; **2021**: 6655185.

273. Chen R, Liang W, Jiang M, et al. Risk Factors of Fatal Outcome in Hospitalized Subjects With Coronavirus Disease 2019&#xa0;From a Nationwide Analysis in China. *CHEST* 2020; **158**(1): 97-105.

274. Du RH, Liu LM, Yin W, et al. Hospitalization and Critical Care of 109 Decedents with COVID-19 Pneumonia in Wuhan, China. *Ann Am Thorac Soc* 2020; **17**(7): 839-46.

275. Yao T, Gao Y, Cui Q, et al. Clinical characteristics of a group of deaths with COVID-19 pneumonia in Wuhan, China: a retrospective case series. *BMC Infectious Diseases* 2020; **20**(1): 695.

276. Mei Q, Wang AY, Bryant A, et al. Survival Factors and Metabolic Pathogenesis in Elderly Patients (≥65) With COVID-19: A Multi-Center Study. *Front Med (Lausanne)* 2020; **7**: 595503.

277. Wenbin L, Qiang Z, Tonghui W. 某2019冠状病毒病救治专科医院抗菌药物使用情况. *中南大学学报:医学版* 2020; **45**(5): 571-5.

278. Hui Z, Bo Y, Hong W, Ming X. 新型冠状病毒肺炎37例死亡原因分析. *广东医学* 2020; **41**(20): 2059-63.

279. Wong CKH, Wan EYF, Luo S, et al. Clinical outcomes of different therapeutic options for COVID-19 in two Chinese case cohorts: A propensity-score analysis. *eClinicalMedicine* 2021; **32**.

280. Yayla BCC, Aykac K, Ozsurekci Y, Ceyhan M. Characteristics and Management of Children With COVID-19 in a Tertiary Care Hospital in Turkey. *Clinical Pediatrics* 2020; **60**(3): 170-7.

281. Zhu L, Wang J, Huang R, et al. Clinical characteristics of a case series of children with coronavirus disease 2019. *Pediatric Pulmonology* 2020; **55**(6): 1430-2.

282. Wenjing C, Peng Z, Xiang L. 抗菌药物在新型冠状病毒肺炎患者临床治疗中的合理使用. *抗感染药学* 2020; **17**(7): 1072-5.

283. Yock-Corrales A, Lenzi J, Ulloa-Gutiérrez R, et al. High rates of antibiotic prescriptions in children with COVID-19 or multisystem inflammatory syndrome: A multinational experience in 990 cases from Latin America. *Acta Paediatr* 2021; **110**(6): 1902-10.

284. Tizkam HH, Fadhil OQ, Ghazy EA. Effect of covid-19 on bacterial resistance. *Systematic Reviews in Pharmacy* 2020; **11**.

## Figure S1. Additional forest plots


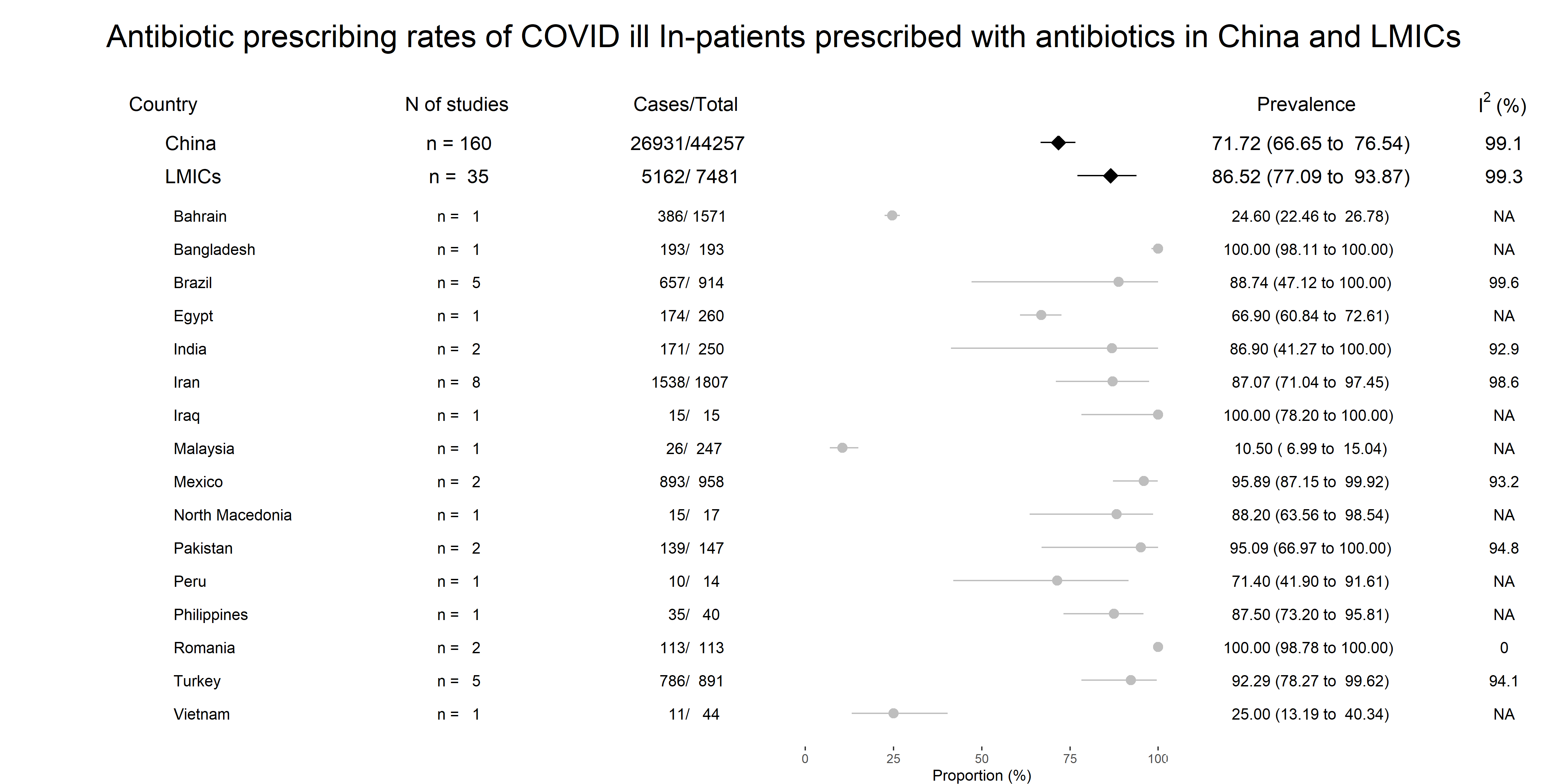


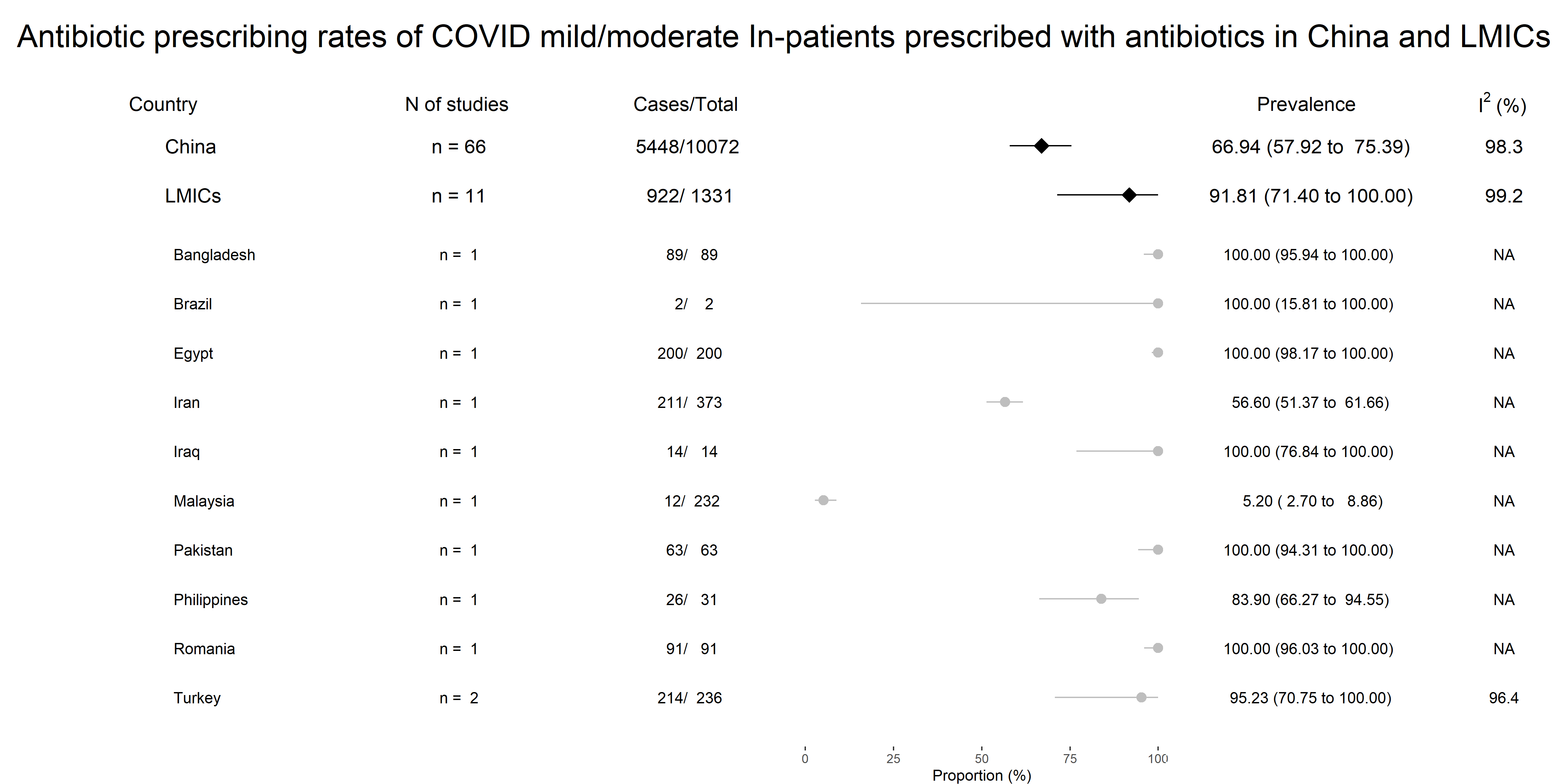


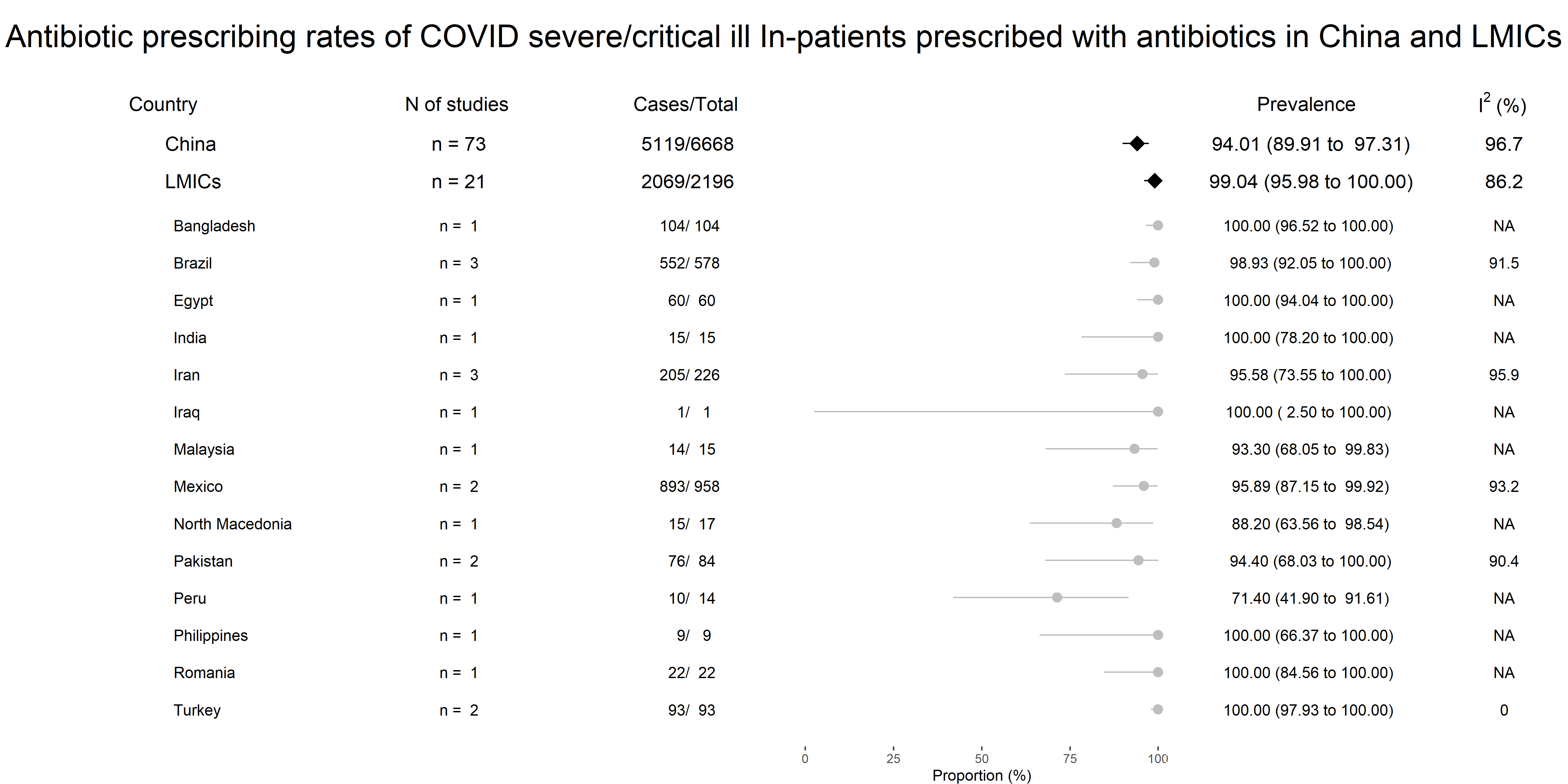


## Table S4. Meta-regression results

Table S4A. Meta-regression of antibiotic prescribing rate in COVID-19 inpatients in China

| **Moderator** | **N study** | **Beta** | **Intercept** | **Lower 95%** | **Upper 95%** | **R^2^** | **P-value** | **I^2^** |
| --- | --- | --- | --- | --- | --- | --- | --- | --- |
| Average %Male | 157 | 0.32 | 0.84 | 0.55 | 1.12 | 0.22 | 0.250 | 99.2 |
| Mean Hospital Stay (day) | 57 | 0.002 | 1.05 | -0.23 | 0.87 | 0.07 | 0.314 | 99.2 |
| %Discharged | 115 | -0.32 | 1.26 | 0.94 | 1.17 | 9.20 | < 0.001 | 98.6 |
| %Mortality | 138 | 0.52 | 0.94 | -0.01 | 0.002 | 14.90 | < 0.001 | 99.0 |

Table S4B. Meta-regression of antibiotic prescribing rate in COVID-19 inpatients in other LMICs

| **Moderator** | **N study** | **Beta** | **Intercept** | **Lower 95%** | **Upper 95%** | **R^2^** | **P-value** | **I^2^** |
| --- | --- | --- | --- | --- | --- | --- | --- | --- |
| Average %Male | 32 | 0.14 | 1.08 | 0.30 | 1.87 | 0.00 | 0.833 | 99.1 |
| Mean Hospital Stay (day) | 16 | -0.03 | 1.39 | -1.18 | 1.46 | 18.50 | 0.041 | 99.2 |
| %Discharged | 20 | -0.15 | 1.27 | 1.02 | 1.77 | 0.00 | 0.619 | 98.6 |
| %Mortality | 29 | 0.35 | 1.08 | -0.05 | 0.00 | 4.42 | 0.147 | 98.9 |

## Table S5. Subgroup analysis

Table S5A. Subgroup analysis of studies in China by study design

| **Test for subgroup differences (random effects):** Q=8.45, DF=5, p-value = 0.1329 | | | | | | | |
| --- | --- | --- | --- | --- | --- | --- | --- |
| **Design** | **N study** | **Cases** | **Total** | **Prevalence (%)** | **Lower 95%** | **Upper 95%** | **I^2^** |
| Cohort | 148 | 25971 | 42828 | 71.4 | 66.2 | 76.3 | 99.1% |
| RCT | 7 | 683 | 956 | 72.3 | 43.6 | 93.4 | 98.6% |
| Case series | 4 | 48 | 64 | 72.9 | 24.0 | 100.0 | 93.8% |
| Case control | 2 | 114 | 306 | 41.1 | 21.6 | 62.1 | 91.6% |
| Cross-sectional | 1 | 168 | 226 | 74.3 | 68.4 | 79.8 | NA |

Table S5B. Subgroup analysis of studies in other LMICs by study design

| **Test for subgroup differences (random effects):** Q=2.23, DF=4, p-value = 0.6943 | | | | | | | |
| --- | --- | --- | --- | --- | --- | --- | --- |
| **Design** | **N study** | **Cases** | **Total** | **Prevalence (%)** | **Lower 95%** | **Upper 95%** | **I^2^** |
| Case series | 2 | 30 | 32 | 94.8 | 77.4 | 100.0 | 52.8% |
| Cohort | 23 | 22413 | 142441 | 83.0 | 69.0 | 93.4 | 99.8% |
| Cross-sectional | 4 | 142 | 404 | 69.9 | 22.3 | 99.4 | 98.9% |
| RCT | 10 | 1343 | 1693 | 88.4 | 74.2 | 97.4 | 98.3% |

Table S5C. Subgroup analysis of studies in China by province (city)

| **Test for subgroup differences (random effects):** Q=312.27, DF=22, p-value < 0.0001 | | | | | | | |
| --- | --- | --- | --- | --- | --- | --- | --- |
| **Province** | **N study** | **Cases** | **Total** | **Prevalence (%)** | **L 95%** | **U 95%** | **I^2^** |
| Anhui | 4 | 221 | 395 | 53.2 | 37.3 | 68.8 | 0.8 |
| Beijing | 6 | 159 | 519 | 30.3 | 17.2 | 45.3 | 0.9 |
| Chongqing | 2 | 79 | 196 | 39.3 | 29.2 | 49.8 | 0.5 |
| Fujian | 1 | 96 | 199 | 48.2 | 41.3 | 55.2 | NA |
| Gansu | 2 | 679 | 2550 | 42.6 | 11.5 | 77.5 | 1.0 |
| Guangdong | 10 | 1125 | 1850 | 73.4 | 52.8 | 89.7 | 1.0 |
| Hainan | 1 | 172 | 457 | 37.6 | 33.2 | 42.1 | NA |
| Hebei | 1 | 47 | 51 | 92.2 | 82.9 | 98.3 | NA |
| Henan | 3 | 196 | 212 | 75.5 | 27.7 | 100.0 | 1.0 |
| Hubei (except Wuhan) | 8 | 751 | 918 | 85.8 | 72.5 | 95.2 | 1.0 |
| Hubei (Wuhan) | 75 | 15321 | 22075 | 81.8 | 75.6 | 87.2 | 1.0 |
| Hunan | 9 | 793 | 1520 | 60.5 | 45.5 | 74.6 | 0.9 |
| Jiangsu | 6 | 636 | 1118 | 59.2 | 31.5 | 84.1 | 1.0 |
| Jiangxi | 2 | 38 | 92 | 40.9 | 19.0 | 64.8 | 0.8 |
| Jilin | 1 | 84 | 93 | 90.3 | 83.4 | 95.6 | NA |
| Liaoning | 1 | 29 | 55 | 52.7 | 39.4 | 65.9 | NA |
| Multiregions | 8 | 5250 | 9387 | 68.1 | 41.5 | 89.5 | 1.0 |
| Ningxia | 1 | 24 | 73 | 32.9 | 22.5 | 44.1 | NA |
| Shaanxi | 1 | 25 | 40 | 62.5 | 46.9 | 77.0 | NA |
| Shandong | 3 | 397 | 569 | 66.4 | 55.3 | 76.6 | 0.6 |
| Shanghai | 4 | 317 | 794 | 40.0 | 32.9 | 47.3 | 0.8 |
| Sichuan | 2 | 63 | 176 | 35.6 | 27.2 | 44.6 | 0.3 |
| Zhejiang | 11 | 482 | 1041 | 60.3 | 34.3 | 83.5 | 1.0 |

*Subgroup analysis was conducted by grouping meta-analysis results of antibiotic prescribing rates in COVID-19 inpatients using Freeman-Tukey Double arcsine transformation and inverse variance meta-analysis approaches in random effects models. Prevalence of antibiotic prescribing and its 95% confidence intervals (CIs) from studies were estimated using the exact method.*

## Figure S2. Forest plots of antibiotic use in COVID-19 patients with pre-existing conditions


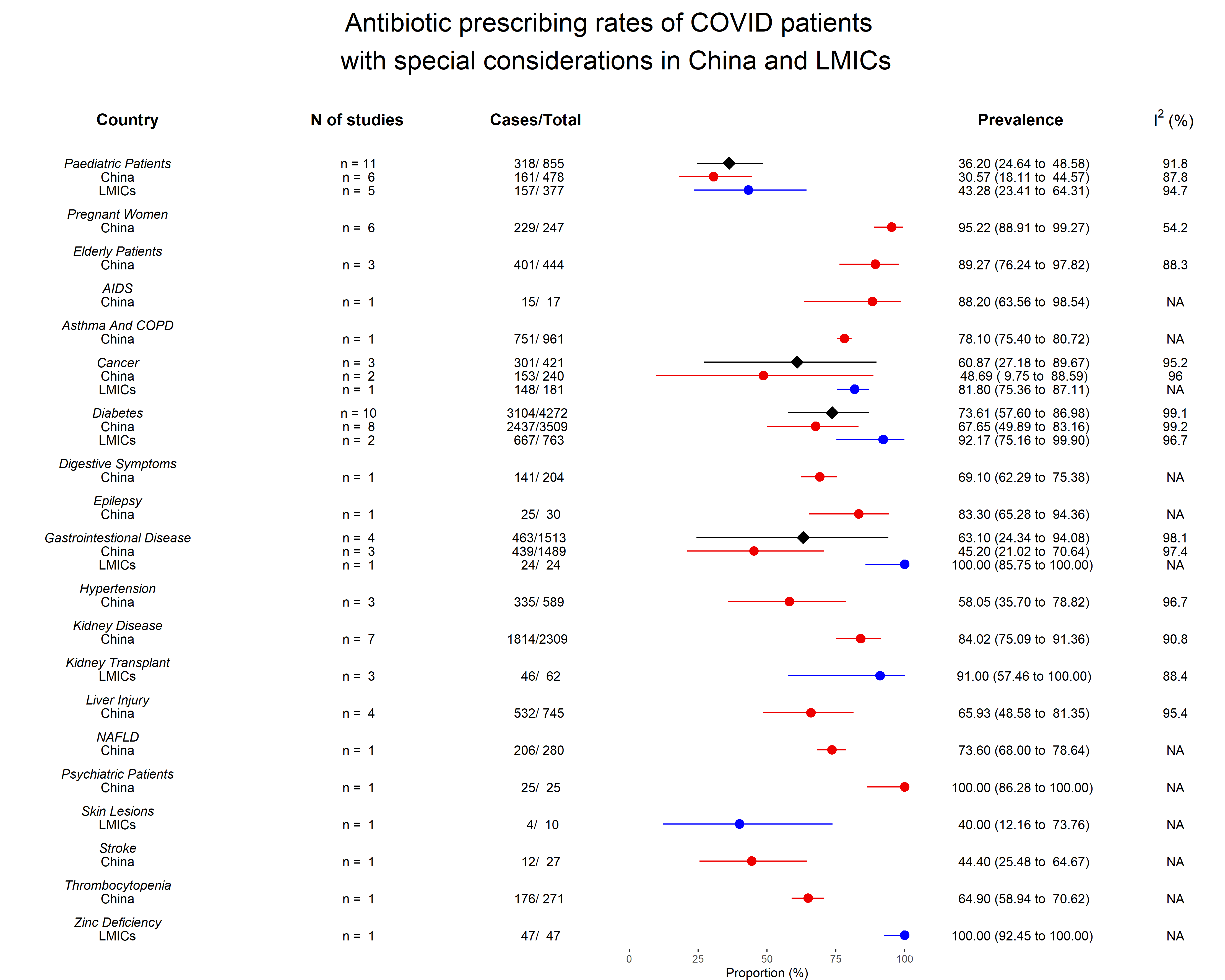


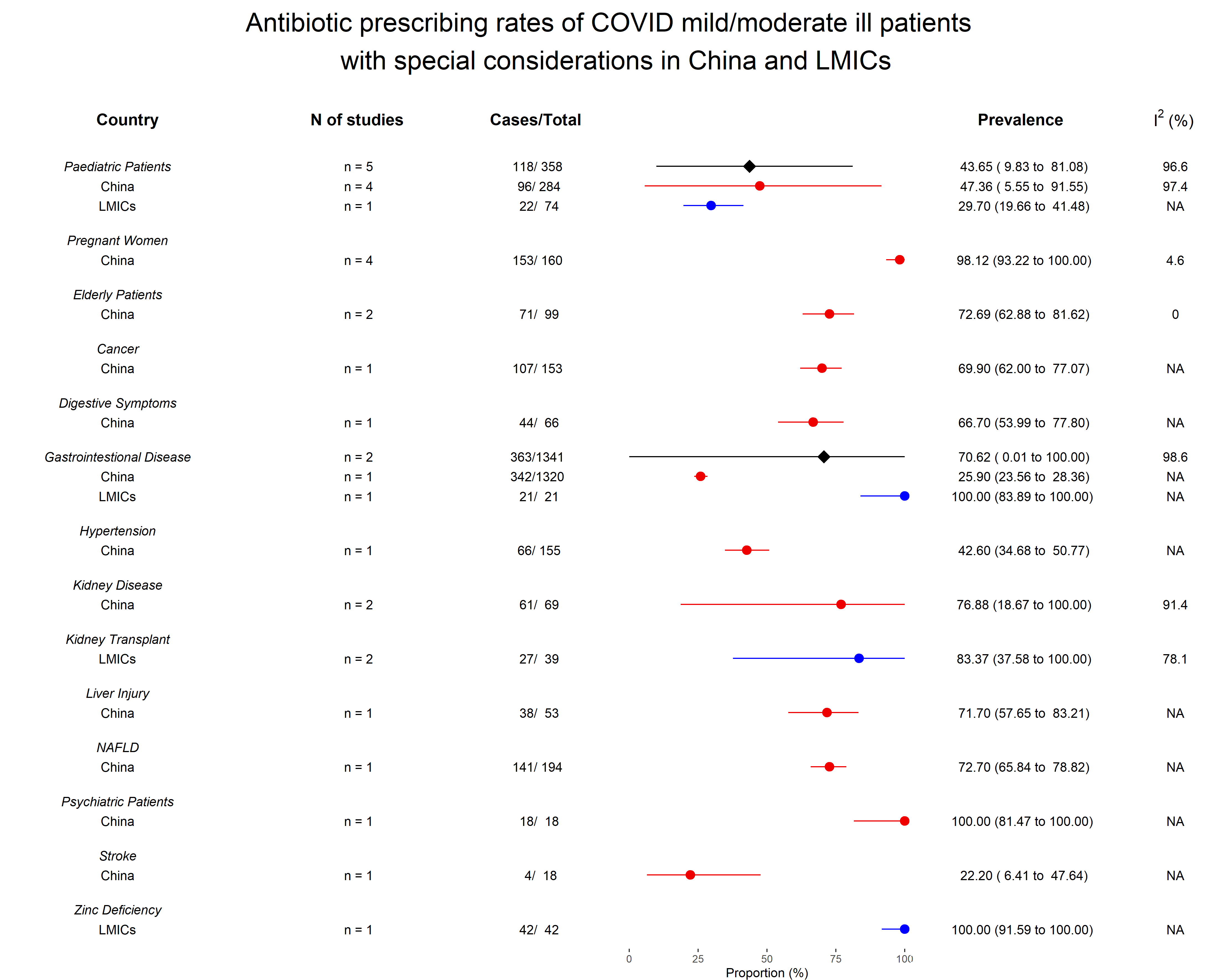


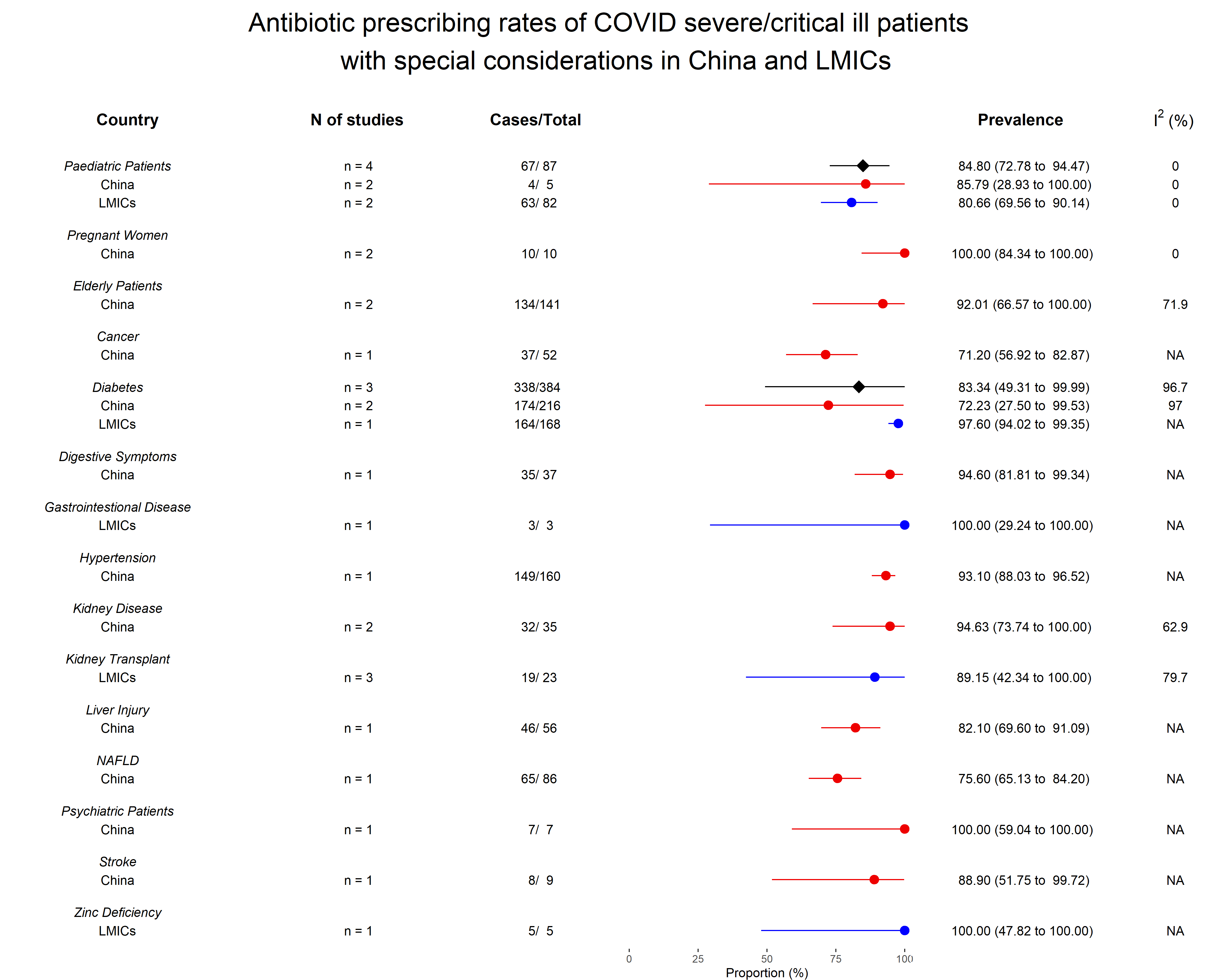


## Table S6. Mortality rates of COVID-19 patients and patients with specific conditions

| **Patient Type** | **Country** | **Study**  **(k)** | **Died/Total**  **(n/N)** | **Mortality**  **Median [IQR]** | **Mortality**  **Mean (95% CI)** |
| --- | --- | --- | --- | --- | --- |
| Inpatients | China | 146 | 3190/39377 | 1.4 [0-11.5] | 7.6 (4.8-11.0) |
|  | Other LMICs | 33 | 1312/9441 | 15.7 [2.1-28.4] | 19.0 (9.8-30.1) |
| Paediatric Patients | China | 6 | 1/478 | 0 [0-0] | 0.3 (0-1.0) |
|  | Other LMICs | 7 | 8/481 | 1.8 [0.6-2.3] | 1.2 (0.1-2.8) |
| Pregnant Women | China | 5 | 0/213 | 0 [0-0] | 0 (0-0.4) |
| Elderly Patients | China | 3 | 218/444 | 58.8 [48.0-59.0] | 50.5 (34.6-66.2) |
| Cancer | China | 2 | 40/240 | 9.8 [4.9-14.6] | 6.9 (0-36.1) |
|  | Other LMICs | 1 | 69/181 | NA | 38.1 (31.0-45.6) |
| Diabetes | China | 7 | 478/3353 | 15.4 [3.6-27.0] | 15.9 (3.4-35.0) |
|  | Other LMICs | 2 | 160/763 | 33.7 [22.3-45.1] | 31.2 (0.6-79.2) |
| Gastrointestinal Disease | China | 2 | 0/1415 | 0 [0-0] | 0 (0-0.01) |
|  | Other LMICs | 1 | 6/24 | NA | 25.0 (9.8-46.7) |
| Hypertension | China | 3 | 22/589 | 3.2 [1.6-4.4] | 2.7 (0.4-6.7) |
| Kidney Disease | China | 7 | 471/2419 | 15.2 [11.6-45.2] | 27.3 (10.8-47.7) |
| Kidney Transplant | Other LMICs | 3 | 15/62 | 20 [16.3-43.3] | 30.0 (3.6-65.5) |
| Liver Injury | China | 4 | 108/745 | 3.6 [2.5-9.0] | 7.0 (1.0-17.1) |

## Table S7. Discharge rates of COVID-19 inpatients and patients with specific conditions

| **Patient Type** | **Country** | **Study**  **(k)** | **Discharged/Total**  **(n/N)** | **Discharge rate**  **Mean/median (IQR)** | **Discharge rate**  **(95% CI)** |
| --- | --- | --- | --- | --- | --- |
| Inpatients | China | 120 | 17415/23092 | 88.9 [55.0-100] | 79.5 (72.5-85.6) |
|  | Other LMICs | 24 | 5116/6614 | 75.9 [53.1-87.9] | 71.1 (56.0-84.3) |
| Paediatric Patients | China | 6 | 474/478 | 100 [99.6-100] | 100.0 (99.5-100.0) |
|  | Other LMICs | 5 | 290/316 | 96.5 [89.9-98.2] | 94.1 (87.0-98.8) |
| Pregnant Women | China | 3 | 80/154 | 13.3 [11.0-39.4] | 28.3 (1.6-67.9) |
| Elderly Patients | China | 3 | 152/444 | 40.8 [33.6-41.0] | 34.5 (23.7-46.2) |
| Cancer | China | 1 | 35/35 | NA | 100.0 (90.0-100.0) |
|  | Other LMICs | 1 | 112/181 | NA | 61.9 (54.4-69.0) |
| Diabetes | China | 4 | 761/1163 | 51.0 [34.4-71.7] | 55.9 (29.8-80.3) |
|  | Other LMICs | 1 | 156/595 | NA | 26.2 (22.7-30.0) |
| Gastrointestinal Disease | China | 2 | 1213/1415 | 64.0 [51.5-76.6] | 66.9 (15.6-99.8) |
|  | Other LMICs | 1 | 18/24 | NA | 75.0 (53.3-90.2) |
| Hypertension | China | 2 | 475/525 | 90.5 [90.5-90.5] | 90.5 (87.8-92.9) |
| Kidney Disease | China | 4 | 505/645 | 69.3 [44.5-83.4] | 59.4 (19.3-93.4) |
| Kidney Transplant | Other LMICs | 2 | 12/22 | 56.7 [45.0-68.3] | 56.8 (13.0-95.4) |
| Liver Injury | China | 2 | 343/480 | 83.3 [76.2-90.4] | 85.5 (49.7-100.0) |

## Table S8. Summary of studies included for bacterial infection analysis

|  | **China** | **Other LMICs** |
| --- | --- | --- |
| Total number of studies (patients) | 18 (4,228) | 7 (3,301) |
| Sample size^*^ | 106 (29 - 1,123) | 595 (19 - 990) |
| Percentage of males^*^ | 63% (60 - 67)^†^ | 58% (NA)^‡^ |
| Patient type |  |  |
| General | 15 (2,984)^≠^ | 5 (1,716) |
| Paediatric patients | 1 (75) | 1 (990) |
| Diabetes | 1 (1,105) | 1 (595) |
| Hypertension | 1 (64) | - |
| Setting |  |  |
| Inpatients | 18 (4,228) | 7 (3,301) |
| Design |  |  |
| Case control | 1 (64) | - |
| Case report/series | - | 2 (615) |
| Cohort | 17 (4164) | 5 (2,686) |
| Infection source |  |  |
| Co-infection | 4 (399) | 2 (279) |
| Secondary infection | 8 (3,222) | 4 (2,032) |
| Not reported | 6 (607) | 1 (990) |

^*^ Median (range); ^†^ k = 2 studies; ^‡^ k = 1 study; ^≠^ Total number of studies (patients)
